# A systematic review of risk factors for mortality among tuberculosis patients in South Africa

**DOI:** 10.1186/s13643-023-02175-8

**Published:** 2023-02-23

**Authors:** Tamaryn J Nicholson, Graeme Hoddinott, James A Seddon, Mareli M Claassens, Marieke M van der Zalm, Elisa Lopez, Peter Bock, Judy Caldwell, Dawood Da Costa, Celeste de Vaal, Rory Dunbar, Karen Du Preez, Anneke C Hesseling, Kay Joseph, Ebrahim Kriel, Marian Loveday, Florian M Marx, Sue-Ann Meehan, Susan Purchase, Kogieleum Naidoo, Lenny Naidoo, Fadelah Solomon-Da Costa, Rosa Sloot, Muhammad Osman

**Affiliations:** 1 Desmond Tutu TB Centre, Department of Paediatrics and Child Health, Faculty of Medicine and Health Sciences, Stellenbosch University, Cape Town, South Africa; 2 Department of Infectious Diseases, Imperial College London, London, United Kingdom; 3 Department of Human, Biological and Translational Medical Sciences, School of Medicine, University of Namibia, Windhoek, Namibia; 4 IS Global, Barcelona Centre for International Health Research (CRESIB), Hospital Clínic-Universidad de Barcelona, Barcelona, Spain; 5 Community Services and Health Directorate, City of Cape Town, Cape Town, South Africa; 6 Division of Medical Microbiology, Department of Pathology, Faculty of Medicine and Health Sciences, Stellenbosch University and National Health Laboratory Service, Tygerberg Hospital, Cape Town, South Africa; 7 Division of Forensic Medicine and Toxicology, Department of Pathology, Faculty of Health Sciences, University of Cape Town, Cape Town, South Africa; 8 Metro Health Services, Southern and Western Substructure, Western Cape Government: Health, Cape Town, South Africa; 9 HIV and other Infectious Diseases Research Unit, South African Medical Research Council, KwaZulu-Natal, Durban, South Africa; 10 Centre for the AIDS Programme of Research in South Africa, CAPRISA-SA-MRC HIV-TB Pathogenesis and Treatment Research Unit, Nelson R Mandela School of Medicine, University of KwaZulu-Natal, Durban, South Africa; 11 DSI-NRF Centre of Excellence in Epidemiological Modelling and Analysis (SACEMA), Stellenbosch University, Stellenbosch, South Africa; 12 School of Human Sciences, Faculty of Education, Health and Human Sciences, University of Greenwich, London, United Kingdom

**Keywords:** TB mortality, Case fatality, Risk factors, Systematic review, South Africa

## Abstract

**Background:**

Tuberculosis (TB)-associated mortality in South Africa remains high. This review aimed to systematically assess risk factors associated with death during TB treatment in South African patients.

**Methods:**

We conducted a systematic review of TB research articles published between 2010 and 2018. We searched BioMed Central (BMC), PubMed®, EBSCOhost, Cochrane, and SCOPUS for publications between January 2010 and December 2018. Searches were conducted between August 2019 and October 2019. We included randomised control trials (RCTs), case control, cross sectional, retrospective, and prospective cohort studies where TB mortality was a primary endpoint and effect measure estimates were provided for risk factors for TB mortality during TB treatment. Due to heterogeneity in effect measures and risk factors evaluated, a formal meta-analysis of risk factors for TB mortality was not appropriate. A random effects meta-analysis was used to estimate case fatality ratios (CFRs) for all studies and for specific subgroups so that these could be compared. Quality assessments were performed using the Newcastle-Ottawa scale or the Cochrane Risk of Bias Tool.

**Results:**

We identified 1995 titles for screening, 24 publications met our inclusion criteria (one cross-sectional study, 2 RCTs, and 21 cohort studies). Twenty-two studies reported on adults (*n* = 12561) and two were restricted to children < 15 years of age (*n* = 696). The CFR estimated for all studies was 26.4% (CI 18.1–34.7, *n* = 13257 ); 37.5% (CI 24.8-50.3, *n* = 5149) for drug-resistant (DR) TB; 12.5% (CI 1.1–23.9, *n* = 1935) for drug-susceptible (DS) TB; 15.6% (CI 8.1–23.2, *n* = 6173) for studies in which drug susceptibility was mixed or not specified; 21.3% (CI 15.3-27.3, *n* = 7375) for people living with HIV/AIDS (PLHIV); 19.2% (CI 7.7–30.7, *n* = 1691) in HIV-negative TB patients; and 6.8% (CI 4.9–8.7, *n* = 696) in paediatric studies. The main risk factors associated with TB mortality were HIV infection, prior TB treatment, DR-TB, and lower body weight at TB diagnosis.

**Conclusions:**

In South Africa, overall mortality during TB treatment remains high, people with DR-TB have an elevated risk of mortality during TB treatment and interventions to mitigate high mortality are needed. In addition, better prospective data on TB mortality are needed, especially amongst vulnerable sub-populations including young children, adolescents, pregnant women, and people with co-morbidities other than HIV. Limitations included a lack of prospective studies and RCTs and a high degree of heterogeneity in risk factors and comparator variables.

**Systematic review registration:**

The systematic review protocol was registered in the International Prospective Register of Systematic Reviews (PROSPERO) under the registration number CRD42018108622. This study was funded by the Bill and Melinda Gates Foundation (Investment ID OPP1173131) via the South African TB Think Tank.

**Supplementary Information:**

The online version contains supplementary material available at 10.1186/s13643-023-02175-8.

## Background

A tuberculosis (TB) death is defined as any death during an episode of TB, regardless of treatment or the underlying cause of death [1]. This may include death before or during TB treatment but does not include death after successful completion of antituberculosis treatment [1]. In 2021, the World Health Organization (WHO) estimated that 10.6 million people developed TB worldwide and approximately 1.6 million TB deaths occurred [2]. This included an estimated 304,000 incident cases of TB and 23,000 HIV-negative and 33,000 HIV-positive TB deaths in South Africa [2].

The End TB strategy aims to reduce the annual number of TB deaths (as reported in 2015) by 75% by 2025 and by 95% by 2035, for a reduction in TB CFR from 15% to 6.5% by 2025 [3]. Interim progress against these ambitious targets is measured by evaluating whether milestones have been reached within particular time frames [3]. By 2019, South Africa had reached milestones set for reducing TB incidence but not for reducing TB CFR [4], and a TB CFR of 19% was estimated for 2020 [2]. Although the proportion of all deaths due to TB in South Africa has declined from 6.5% in 2016 to 6% in 2018, TB remains the leading cause of death in South Africa [5].

In a systematic review of risk factors for mortality in people on TB treatment globally between 1966 and 2010, two distinct epidemics dependent on TB/HIV burden were described [6]. In high TB/HIV regions HIV positivity, the presence of atypical changes on X-ray, sputum smear-negative disease, advanced immunosuppression, and malnutrition were identified as risk factors for mortality. In settings with low TB/HIV prevalence, increased age, typical features of severe TB on chest radiograph, smear positivity and low socioeconomic status were identified as risk factors [6]. In South Africa, a country with a high burden of TB, HIV, and DR-TB, individual studies have identified co-infection with HIV [7–12]; failing to start treatment following a TB diagnosis [13]; undiagnosed TB (found at post mortem) [14, 15]; drug resistance [11, 16–18]; and co-morbidities like diabetes [19] as important risk factors for TB mortality. However, there is no comprehensive review of the risk of TB mortality or the relative importance of risk factors for TB mortality in South Africa. National TB policymakers have prioritised understanding TB mortality in South Africa and the analysis of data from 2010 onwards.

We aimed to identify risk factors for TB mortality during TB treatment in South Africa for patients with DS-TB, DR-TB, with and without HIV, and for adult and paediatric populations.

## Methods

### Identification and selection of papers

This systematic review is reported according to PRISMA guidelines (Additional file 1: PRISMA 2020 Checklist) [20–22]. An initial search using “Tuberculosis” AND [“treatment outcomes” OR “death” OR “mortality” OR “poor outcome”] AND [“risk factors” OR “determinants” OR “predictors” OR “contributing factors”] AND [“South Africa” OR “Southern Africa” OR “Sub-Saharan Africa” OR “Africa”] was run across BioMed Central (BMC), PubMed®, EBSCOhost, Cochrane, and SCOPUS for publications between January 2010 and December 2018. Searches were conducted between August 2019 and October 2019.

Titles were screened and duplicates identified and removed. Unique titles were reviewed, and studies excluded if they did not investigate risk factors for mortality in TB patients, were in vitro or animal studies, or were review, editorial, opinion, comment, response, or other non-data driven article-types. Concurrent work evaluating mortality using data from a comprehensive programmatic dataset of South African TB treatment registers was in progress at the time of the review [23, 24] and to prevent duplication of findings from the same data sources, studies based on the routine programmatic TB registers (ETR.Net or EDRWeb) were excluded from our review. During the abstract review process, two reviewers excluded further articles based on initial exclusion criteria used for screening titles: mortality not being a defined outcome for the study, a focus on all-cause mortality instead of mortality associated with TB, very narrow sampling criteria, or research designs other than clinical cohort, cross-sectional, or case comparison. We did not set any exclusion criteria for timing of death, before or during TB treatment. Studies were grouped for synthesis by subgroup and TB mortality risk factors to determine comparability.

### Data abstraction and analysis

We conducted data abstraction via online survey using Research Electronic Data Capture (REDCap) [25]. Data included year of publication; TB drug susceptibility test (DST) pattern; eligible population; age groups; sampling strategy; sample size; definition of TB death or CFR; TB treatment and duration; risk factors for death including effect measures and 95% confidence intervals (CI); and the limitations of the study (full list presented in Additional file 1: Complete list of outcomes and variables for which data were sought). Each article was independently reviewed by two of 23 researchers, and any disagreement was resolved in discussion with the principal investigator.

Data extracted were tabulated and subgroups and comparability of risk factors assessed. Due to heterogeneity in effect measures and risk factors evaluated, a formal meta-analysis of risk factors for TB mortality was not appropriate. We conducted a random effects meta-analysis using the restricted maximum likelihood estimation method to establish CFRs for all studies and for specific subgroups to contribute to our understanding of mortality risk factors; however, this was not an original aim of the study. One author assessed the quality of evidence for case control and cohort studies using the Newcastle-Ottawa scale (NOS) [26] and used the Cochrane Risk of Bias Tool [27] to assess the quality of randomised control trials (RCTs).

## Results

### Literature search results

**Fig. 1:**
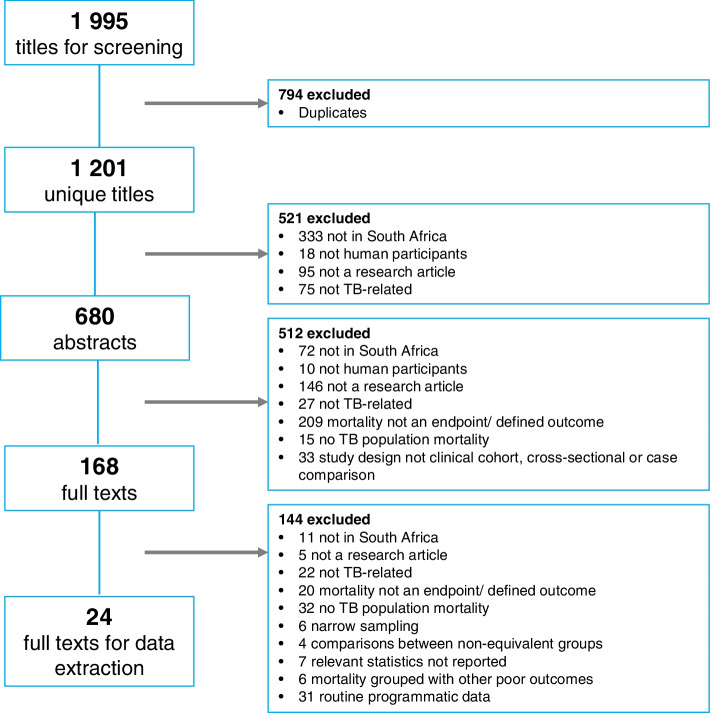
Flow diagram outlining the selection of studies included in systematic review

We identified 1995 titles for screening and 24 publications met our inclusion criteria (Fig. 1). Study designs included retrospective studies (*n* = 13), prospective cohort studies (*n* = 8), two RCTs and one case control study.

### Methodological quality of studies included

Based on the NOS, most cohort studies (*n* = 21) rated well overall, as well as for selection criteria, comparability, and outcomes. However, in relation to loss to follow-up (LTFU), seven studies either reported attrition greater than 10% without demonstrating a lack of systematic difference between those followed and those LTFU or did not report the number of those LTFU (Additional file 1: Table 1). The two RCTs performed well overall and were rated as low risk, but a lack of blinding was a possible source of bias for both (Additional file 1: Table 2). The case control study scored six out of a maximum of eight points (Additional file 1: Table 3).

**Table 1:** Studies included the TB mortality review ranked by case fatality ratio, 2010–2018 (*n* = 24)

First author and design	Year ^a^	DST pattern	Study location	Study population	Sample size	Deaths	CFR ^b^ (%)	Main risk factors identified (effect measure)
Pietersen [17] *Prospective Cohort*	2014	XDR	Western sub-district, Cape Town, Western Cape; Upington, Northern Cape; Johannesburg, Gauteng.	Adult XDR-TB patients	107	78	72.9	HIV status; ART; gender; net conversion; net reversion; age at diagnosis; drugs in treatment regimen; mixed ancestry (HR)
Gandhi [18] *Case Control*	2012	MDR, XDR	Tugela Ferry, KwaZulu-Natal	HIV+ adult MDR- and XDR-TB patients	262	189	72.1	CD4 count; drug resistance pattern; ART status (HR)
Kvasnovsky [28] *Retrospective Cohort*	2011	XDR	Port Elizabeth, Eastern Cape	TB patients starting treatment	274	160	58.4	Age; weight; bilateral cavity disease; smear; previous TB treatment; status; year; ART status (OR)
Pietersen [29] *Retrospective Cohort*	2015	XDR	Western Cape and Northern Cape	Adults (≥ 18) with genotyped isolates	178	93	52.2	Weight at diagnosis; HIV status; Capreomycin resistance; TB drugs (Capreomycin, Moxifloxacin; Co-amoxicillin/clavulanic acid) (OR)
Marais [30] *Retrospective Review*	2011	DS, MDR	Manenberg, Cape Town, Western Cape	Adults with CSF-confirmed TBM.	120	59	49.2	CD4; British Medical Research Council TBM disease grade (OR)
O'Donnell [31] *Retrospective Cohort*	2013	XDR	KwaZulu-Natal	XDR TB adult (> 18 years of age)	114	48	42.1	Gender; previous TB treatment; HIV status; culture conversion within 2 months; ART (HR)
Dheda [32] *Retrospective Cohort*	2010	XDR	Western Cape, Eastern Cape, Northern Cape, Gauteng	Adults (≥ 16)	199	83	41.7	Regimen; previous TB treatment; HIV (HR)
Olaleye [33] *Retrospective Cohort*	2016	MDR	Witbank, Mpumalanga	Adults	442	151	34.2	Smear; HIV status; gender; age (HR)
Janssen [34] *Prospective Cohort*	2017	DS	Khayelitsha, Cape Town, Western Cape	HIV+ adults with CD4+ < 350	60	16	26.7	Mycobacteremia; treatment delay (HR)
Umanah [35] *Retrospective Review*	2015	MDR	Johannesburg, Gauteng	HIV+ adults (≥ 18) alive > 24 h of initiating Rx.	947	181	19.1	Weight; TB location; regimen; age; sex; CD4; co-morbidity; adverse events; other opportunistic infections; cavitary radiograph changes (OR)
Marais [36] *Retrospective Review*	2014	MDR	Johannesburg, Gauteng	MDR TB patients in hospital	351	65	18.5	Diagnosis year; gender; TB strain; age (OR)
Brust [37] *Retrospective Cohort*	2010	MDR	Durban, KwaZulu-Natal	Minimum age not specified (9.6% reported < 20 years old).	1 209	223	18.4	HIV status; previous TB treatment; mono-resistance (Ethambutol); year (OR)
Pepper [38] *Prospective Cohort*	2011	DS, MDR	Cape Town, Western Cape	HIV+ adults eligible for ART at TB diagnosis	100	15	15	CD4+ count, 100 cells/mL (OR)
Lawn [39] *Prospective Cohort*	2017	DS, RIF mono	Klipfontein, Cape Town, Western Cape	HIV+ adults (≥ 18)	139	19	13.7	u-LAM; gender; mono-resistance (HR)
Griesel [40] *Prospective Cohort*	2018	NS	Cape Town, Western Cape	Hospitalised HIV+ adults (≥ 18), cough and one WHO danger sign	255	34	13.3	Respiratory rate; temperature; patient-ambulance; HIV status; BMI; ART; hypotension; confusion (OR)
Field [41] *Retrospective Review*	2014	NS	North West Province (Platinum Mine)	Adult miners	4 162	509	12.2	Diagnosis age; diagnostic certainty; HIV/ART status; TB location (IRR)
Kerkhoff [42] *Prospective Cohort*	2016	NS	Cape Town, Western Cape	HIV+ adults; ART naïve or newly diagnosed	174	21	12.1	Hepcidin level; HIV status-CD4; HIV status-viral load; ambulation (HR)
Kendon [43] *Retrospective Review*	2012	NS	Durban, KwaZulu-Natal	HIV+ adult in-patients on TB therapy for HIV-associated TB and adults on TB therapy at ART initiation	458	55	12.0	ART timing; renal impairment; inpatient initiation; TB site (HR)
Brust [44] *Prospective Cohort*	2018	MDR	KwaZulu-Natal	Adults (≥ 18)	206	24	11.7	Age; HIV status; previous TB treatment, chest radiograph; gender; CD4 (HR)
Loveday [45] *Prospective Cohort*	2012	MDR	KwaZulu-Natal (Specialised TB hospital, 4 MDR sites)	MDR TB adults (≥ 18);	860	97	11.3	Site; age (OR)
Seddon [46] *Retrospective Cohort*	2012	DS, MDR, INH mono, RIF mono	Cape Town, Western Cape	Children under 13 years confirmed or probable TBM.	123	11	8.9	MDR TB; HIV status (OR)
Abdool Karim [47] *Randomised Control Trial*	2010	NS	Durban, KwaZulu-Natal	Adult smear+ HIV+ with CD4+ counts < 500 cells/mm^3^	642	52	8.1	Integrated vs sequential ART (HR)
Churchyard [48] *Randomised Control Trial*	2011	DS	Free State	Adult miners	1 302	104	8.0	Screening-timing at 6 and 12 months (HR)
Yotebieng [49] *Retrospective Cohort*	2010	DS	Soweto, Johannesburg, Gauteng	ART-naive HIV+ children initiated on TB treatment	573	37	6.5	HIV status; ART timing; weight (HR)

*ART* antiretroviral therapy, *CFR* case fatality ratio, *CSF* cerebrospinal fluid, *DR* drug-resistant, *DS* drug-susceptible, *DST* drug-susceptible test, *HR* hazard ratio, *INH* isoniazid, *IRR* incident rate ratio, *MDR* multidrug-resistant, *Mono* mono-resistant, *NS* non-specified, *OR* odds ratio, *RMR* rifampicin mono-resistant, *RR* relative risk, *TB* tuberculosis, *TBM* tuberculosis meningitis, *u-LAM* urinary lipoarabinomannan, *XDR* extensively drug-resistant; *+* positive, − negative

^a^Year refers to the year of publication

^b^Case fatality ratio calculated as the number of deaths as a proportion of the total sample size

**Table 2:** Frequency of reported key diagnostic and treatment variables among included studies, South Africa, 2010–2018 (*n* = 24)

	Number of studies	**%**
Method of TB diagnosis		
Clinical	6	25.0
Chest radiograph	5	20.8
Laboratory ^a^	24	100.0
- Smear	10 ^a^	41.7
- Culture	21 ^a^	87.5
- Xpert	6 ^a^	25.0
Not reported	1 ^b^	4.2
Other	2	8.3
Treatment regimen described	14	58.3
Place of death reported	2	8.3
Duration (timing)		
- Between diagnosis and death	4	16.7
- Between treatment initiation and death	9	37.5
- Of treatment	13	54.2

*TB* tuberculosis

^a^Laboratory diagnosis is a subset of method of TB diagnosis. Some studies reported performing more than one type of laboratory diagnosis. The sum of these diagnostics therefore exceeds the total number of studies reported

^b^One study included 23% (959/4162) of TB patients with no data on the method of diagnosis [41]

**Table 3:** Tuberculosis case fatality ratios by specific population and reported subgroups, South Africa, 2010–2018

Specific population	First author and year published	Total study population	Deaths	CFR	Subgroup	n	Deaths	CFR	Unknown outcome	CFR^g^ known outcomes
DS-TB, HIV+, children	Yotebieng 2010 [49]	573	37	6.5	Death before ART	112	7	6.3	38	9.5
					Death after ART	461	30	6.5	37	7.1
DST mixed or not specified, HIV+	Kerkhoff 2016 [42]	174	21	12.1	Hospitalised	116	12	10.3	8	11.1
					Ambulatory	58	9	15.5	1	15.8
DST mixed or not specified, HIV+	Griesel 2018 [40]	255	34	13.3						
DS-TB, HIV+	Janssen 2017 [34]	60	16	26.7						
DST mixed or not specified, HIV+	Abdool Karim 2010 [47]	642	52	8.1	Integrated ^a^ ART	429	25	5.8		
					Sequential ^a^ ART	213	27	12.7		
DS-TB	Churchyard 2011 [48]	1 302	104	8.0	Miners: 6-month screening ^b^	670	44	6.6		
					Miners: 12-month screening ^b^	632	60	9.5		
DST mixed or not specified	Field 2014 [41]	4 162	509	12.2	HIV− or unknown	1 341	37	2.8		
					HIV+ on ART	675	106	15.7		
					HIV+ no ART	2 169	366	16.9		
DST mixed or not specified, HIV+	Kendon 2012 [43]	458	55	12.0	Immediate ^c^ ART	303	47	15.5		
					Early ^c^ ART	85	7	8.2		
					Delayed ^c^ ART	70	1	1.4		
DR-TB	Pietersen 2015 [29]	178	93	52.3	Capreomycin rrs A1401G mutation	154	78	50.6		
					Capreomycin rrs wild type	24	15	62.5		
DR-TB	Kvasnovsky 2011 [28]	274	160	58.4	Treatment started	206	95	46.1		
					HIV− started treatment ^d^	87	31	35.6		
					HIV+ started treatment ^d^	108	55	50.9		
DR-TB	Dheda 2010 [32]	199	83	41.7	Total cohort	199	83	41.7	4	42.6
					On XDR treatment	174	62	35.6		
					HIV+ on XDR treatment	82	34	41.5		
					HIV− on XDR treatment	92	28	30.4		
DR-TB, HIV+	Umanah 2015 [35]	947	181	19.1	HIV+ with MDR	947	181	19.1	250	26.0
					HIV+ ART before TB treatment	545	119	21.8	131	28.7
					HIV+ ART after TB treatment	402	62	15.4	119	21.9
DR-TB	Olaleye 2016 [33]	442	151	34.2	Age 15–60	431	144	33.4		
					Age 60–68	11	7	63.6		
					Men	252	79	31.3		
					Women	190	72	37.9		
					Mpumalanga resident ^d^	196	19	9.7		
					Other province resident ^d^	214	13	6.1		
					Unmarried ^d^	87	22	25.3		
					Married ^d^	57	31	54.4		
					Previous treatment ^d^	89	26	29.2		
					No previous treatment ^d^	345	123	35.7		
					HIV−	43	13	30.2		
					HIV (not tested)	227	70	30.8		
					HIV+	172	68	39.5		
					On ART ^d^	136	53	39.0		
					No ART ^d^	22	7	31.8		
					Smear – ^d^	247	53	21.5		
					Smear + ^d^	183	90	49.2		
DR-TB	Pietersen 2014 [17]	107	78	72.9	General	107	78	72.9	11	81.3
DR-TB	Brust 2018 [44]	206	24	11.7	HIV+	150	19 ^e^	12.7	27 ^e^	15.4
					HIV−	56	3 ^e^	5.4	9 ^e^	6.4
DST mixed or not specified	Marais 2014 [36]	351	65	18.5	Total cohort	351	65	18.5	128	29.1
					HIV− ^d^	72	11	15.3	19	20.8
					HIV+ ^d^	203	45	22.2	53	30.0
					HIV unknown ^d^	49	9	18.4	29	45.0
DR-TB	Gandhi 2012 [18]	262	189	72.1	MDR patients	123	78	63.4		
					XDR patients	139	111	79.9		
DR-TB	Loveday 2012 [45]	860	97	11.3	General	860	97	11.3	212	15.0
					Site–centralised hospital	441	30	6.8	133	9.7
					Site–decentralised	419	67	16.0	79	19.7
DR-TB	O'Donnell 2013 [31]	114	48	42.1	General	114	48	42.1	19	50.5
DR-TB	Brust 2010 [37]	1209	223	18.4	General	1209	223	18.4	252	23.3
					HIV+	362	90	24.9	81	32.0
					Previous TB	959	160	16.7		
HIV+, TBM	Marais 2011 [30]	120	59	49.2	Total cohort	120	59	49.2	14	49.2
					HIV+	106	39	36.8		
DST mixed or not specified, HIV+	Lawn 2017 [39]	139	19	13.7	u-LAM negative ^f^	83	6	7.2		
					u-LAM positive ^f^	53	13	24.5		
DST mixed or not specified, HIV+	Pepper 2011 [38]	100	15	15.0						
DST mixed or not specified, children, TBM	Seddon 2012 [46]	123	11	8.9						

*ART* antiretroviral therapy, *CFR* case fatality ratio, *DR* drug-resistant, *DS* drug-susceptible, *INH* isoniazid, *IRR* incident rate ratio, *MDR* multidrug-resistant, *MR* mono-resistant, *NS* non-specified, *RIF* rifampicin, *TB* tuberculosis, *TBM* tuberculosis meningitis, *u-LAM* urinary lipoarabinomannan, *XDR* extensively drug-resistant, + positive, − negative

^a^The timing of ART initiation defined relative to TB treatment as: Integrated: where ART started within 4 weeks of starting TB treatment, or within 4 weeks of completing the intensive phase of TB treatment; and Sequential: ART: where ART was started within 4 weeks of completion of TB treatment [47]

^b^Screening: All miners were routinely screened for TB using a miniature screening chest radiograph (100 mm × 100 mm)

^c^The timing of ART initiation defined relative to TB treatment as: Immediate: TB patients who started ART ≤ 28 days after starting TB treatment; Early: TB patients who started ART 29–56 days after starting TB treatment; Delayed: TB patients who started ART ≥ 57 days after starting TB treatment [43]

^d^For these subgroups the unknown categories were not specified, and the sum of the subgroups does not equal to the study sample

^e^2 patients who died after cure were not classified by HIV status and could not be reported in each subgroup; and 2 patients who moved had unknown outcomes which were not disaggregated by HIV status

^f^3 patients did not have a u-LAM result

^g^Case fatality ratio calculated using deaths as a proportion of TB patients with a known outcome

### Study characteristics

There was heterogeneity in effect measures with risk factors reported as hazard ratios (HRs), incidence rate ratios (IRR), odds ratios (ORs), and risk ratios (RRs). Twelve studies reported exclusively on DR-TB, and this accounted for 39% of all patients (5149/13257). Of the three studies reporting exclusively on DS-TB, one was of miners and one of children. The remaining nine studies included four on patients with mixed DST patterns and five where the DST was not specified. Only two studies represented children, and these were restricted to very specific populations. The first was of children (defined as 0–13 years) with TB meningitis (TBM) [46] and the second of children (defined as 0–15 years) living with HIV but not on ART [49]. Nine of the studies were restricted to PLHIV and 14 of the remaining 15 studies included HIV as a variable. Only single studies evaluated factors like renal impairment [43], bilateral cavitary disease [28], and adverse events [35] (Table 1).

**Fig. 2:**
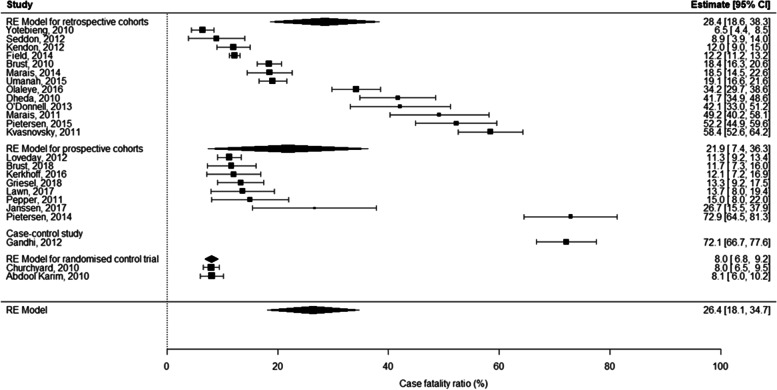
Results of a random effects meta-analysis for case fatality ratios across all studies, South Africa, 2010–2018

**Fig. 3:**
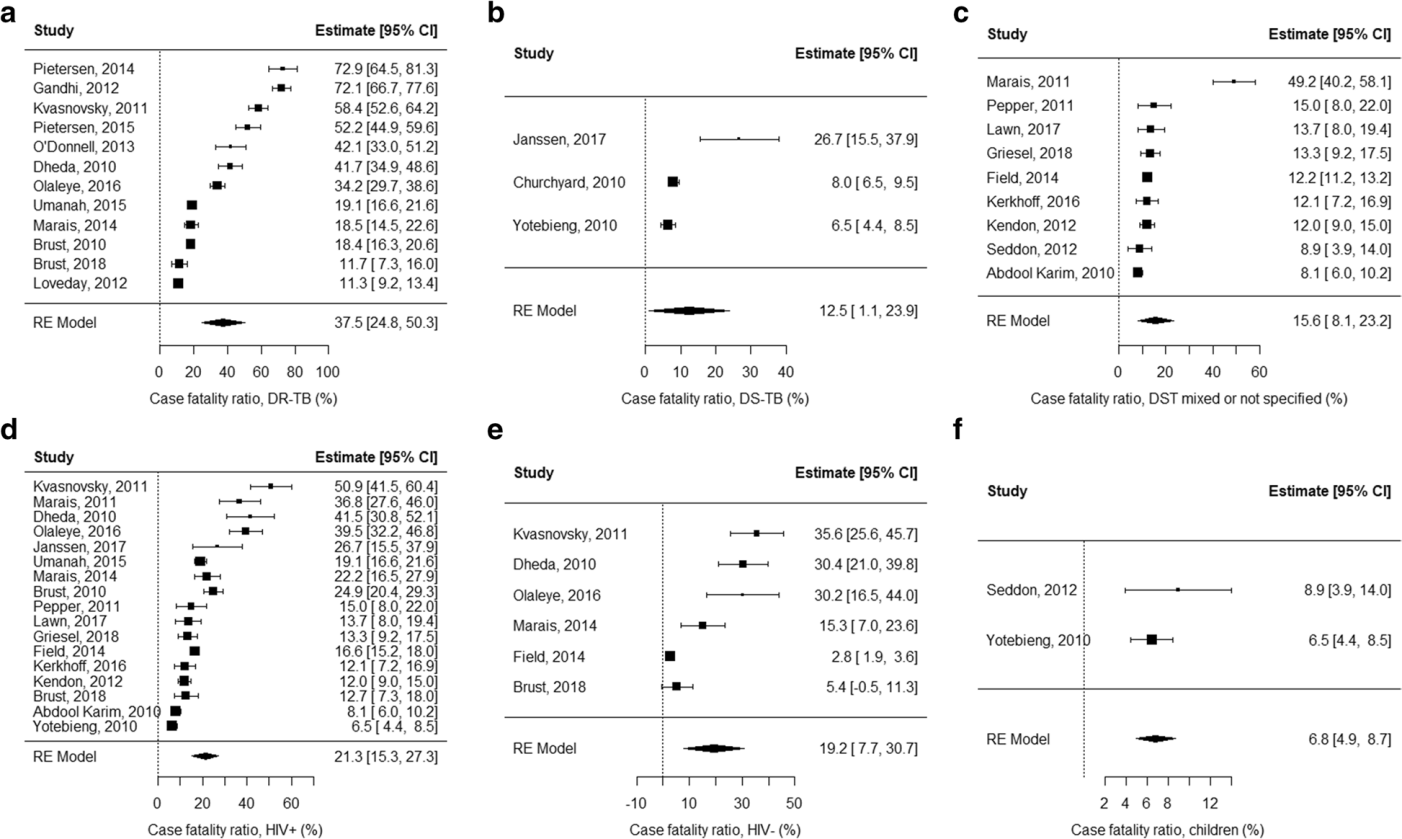
Results of a random effects meta-analysis for case fatality ratios of specific populations in South Africa, 2010–2018, with Panel **A**: drug-resistant TB, Panel **B**: drug-susceptible TB, Panel **C**: drug susceptibility mixed or not specified, Panel **D**: HIV+, Panel **E**: HIV- (Note: Five of the six studies reported on drug-resistant TB, elevating the case fatality ratio for this group), Panel **F**: children

Reporting of TB diagnosis, treatment regimens and time to death across studies was inconsistent: all studies reported some element of laboratory confirmation, but this varied with the reporting of smear, culture, and GeneXpert MTB/RIF assay (Xpert; Cepheid, Sunnyvale, CA); 14 studies described treatment regimens; 13 the duration of treatment; 9 time from treatment initiation to death; 4 time from diagnosis to death; and 2 reported place of death (Table 2). The CFR estimated for all studies was 26.4% (CI 18.1–34.7, *n* = 13257); 37.5% (CI 24.8–50.3, *n* = 5149) for DR-TB; 12.5% (CI 1.1–23.9, *n* = 1935) for DS-TB; 15.6% (CI 8.1–23.2, *n* = 6173) for studies in which drug susceptibility was mixed or not specified and 6.8% (CI 4.9–8.7, *n* = 696) in paediatric studies. There was little difference in CFRs estimated for PLHIV (21.3%, CI 15.3–27.3, *n* = 7375) and HIV-negative TB patients (19.2%, CI 7.7–30.7, *n* = 1691); this is because five of six studies reporting on HIV-negative patients were restricted to DR-TB patients, elevating the CFR for this subgroup (Figs. 2 and 3).

### Case fatality ratios

In the three studies reporting on individuals with DS-TB, 67% (1302/1935) of patients were included from a single study of adult miners [48] and 33% (633/1935) were PLHIV. The highest CFR (26.7%, 16/60) was reported during hospitalisation for PLHIV with CD4 counts < 350 cells/mm^3^ and included death due to suspected bacterial sepsis and disseminated TB [34]. In that study, all patients had started TB treatment within a median of 1 day from diagnosis and 31% of TB patients who died were on ART [34]. In the single study of children with DS-TB the CFR was 6.5% and included seven children with HIV who died prior to initiating antiretroviral therapy (ART). These children died between 2004 and 2008 prior to universal ART access [49] (Table 3).

Twelve studies reported on individuals with DR-TB studies (*n* = 5149), the lowest CFRs were reported for a small sample of HIV-negative patients (CFR = 5.4%, 3/56) [44] and those receiving treatment at centralised hospital sites (CFR = 6.8%, 30/441) [45]. The highest CFR was reported for a cohort of people with extensively drug-resistant (XDR) TB diagnosed between 2002 and 2008 after 5 years of follow up (CFR = 72.9%, 78/107; Table 3) [17]. In studies restricted to PLHIV (*n* = 9) the highest CFRs were reported for a small sample of patients who had a positive urinary lipoarabinomannan (u-LAM) test (CFR = 24.5%, 13/53) [39] and patients with DR-TB who were on ART prior to initiating TB treatment (CFR = 21.8%, 119/545) [35]. The factors associated with low CFRs were the same as those noted in studies of DS-TB; with ART initiated during TB treatment associated with a lower CFR of 5.8% (25/429) [47] (Table 3). In the two studies restricted to children, the CFR in those with TBM was 8.9% (11/123) [46], and 6.3% (7/112) [49] among children with HIV, prior to ART initiation (Table 3). Unknown TB treatment outcomes were reported in 10 studies. CFRs were higher when restricted to TB patients with known outcomes (Table 3).

### Timing of mortality

Nine studies described the time to death from treatment initiation [17, 28, 31, 33–35, 41, 45, 49]. Where time was reported continuously, Kaplan-Meier survival curves were shown over 12 [28] or 24 months [31] and in three studies the median time to death was reported for specific groups [35, 45, 49]. Among 573 ART-naïve children with HIV, treated for TB between 2004 and 2008, 37 children died after a median of 62 days of TB treatment, including seven children who died before ART initiation and 30 who died after ART initiation [49]. Where DR-TB treatment was provided between 2008 and 2009 in different settings, the median time to death was 43 days for those treated at centralised sites compared to 85 days for those treated at decentralised sites [45]. Considering the timing of ART in patients with multidrug-resistant (MDR)-TB treated between 2007 and 2010, the median time to death for those on ART prior to TB treatment was 139 days compared to 321 days for those starting ART after TB treatment [35]. Where time to death from treatment initiation was classified categorically, this varied with deaths reported per month of treatment [41] or at varied time points [17, 33, 34] after starting TB treatment. Survival curves were reported in three studies and time periods varied from 3 months to 3 years [18, 39, 44]. When analysing subgroups, a median survival from diagnosis of 42 days for patients with MDR-TB and 19 days for patient with XDR-TB was reported for patients treated between 2005 and 2006 [18].

### Risk factors for TB mortality

We broadly characterised risk factors as demographic, baseline clinical characteristics, TB disease-related, TB treatment-related and HIV-related. However, factors falling into these categories were varied, some being unique to studies focusing on a sub-population (Additional file 1: Tables 4-7).

#### Demographic factors

Age was referenced in eight studies [17, 28, 33, 36, 41, 44, 45, 49]. The individuals with the highest risk for death were 25–42-year-old adults with XDR-TB, as compared to individuals < 25 years old with XDR-TB (aOR 3.5, 95% CI 1.3–9.6) [28]. Sex was evaluated in eight studies, but results were mixed and not significant [17, 18, 33, 35, 36, 39, 44, 46] (Additional file 1: Table 4).

#### Clinical factors

Weight was evaluated in three studies. Lower weight at TB diagnosis decreased the odds of survival among TB patients with XDR-TB [28]; being underweight (BMI 16–18.49 kg/m^2^) or severely underweight (BMI < 16 kg/m^2^) compared to those with a normal BMI (BMI 18.5 kg/m^2^–24.9 kg/m^2^) increased the odds of mortality among adults with DR-TB [35]; and in ART-naive children with HIV being severely underweight (weight-for-age *Z*-score <− 3) was associated with greater hazard of mortality [49] (Additional file 1: Table 5). A history of previous TB was reported as a risk factor for mortality in four studies [28, 30, 32, 41]. The greatest risk was reported for individuals with XDR-TB who had previously received MDR-TB treatment compared with those that had not (aHR 5.2, 95% CI 1.9–14.1) [32]. In a study restricted to South African miners, one or more previous TB episodes was associated with higher risk of mortality in the first month of treatment (aIRR 2.2, 95% CI 1.5–3.3) and in months 2–6 of treatment (aIRR 1.5, 95% CI 1.2–1.8) [41]. Specific elements of the diagnosis of TB were assessed as risk factors in eight studies [18, 28, 33, 35, 39, 41, 44, 48], six of which reported significant results. The greatest effect was in a group of South African miners treated for TB with microbiological data, where a ‘possible’ TB diagnosis (defined as a negative culture and/or smear) was associated with a greater risk of death within the first month of TB treatment compared to those with a ‘confirmed’ TB diagnosis (defined as a positive culture) (aIRR 6.3, 95%CI 3.2–12.4) [41]. Patients with positive uLAM results compared to those with negative results had a greater risk of death (aHR 4.2, 95% CI 1.5–11.8) [39] and adults with sputum smear-positive DR-TB were at increased risk compared to those with smear-negative disease (HR 3.3, 95% CI 2.1–5.6). Drug resistance was evaluated as a risk factor in four studies [18, 29, 39, 46]. The greatest risk associated with mortality was reported in a study of children with culture confirmed TBM where children with DR-TB had a higher risk for mortality compared to those with DS-TB (aOR 63.9, 95% CI 4.8–843.2) [46]. In a study of TB patients with MDR- and XDR-TB between 2005 and 2006, resistance to more drugs was associated with an increased hazard of mortality [18]. Site of disease was associated with risk of mortality in two studies, both of which reported lower risk in those with extrapulmonary TB when compared to those with pulmonary TB alone [41, 43]. Additionally, in individual studies TB molecular genotypes [36] and the setting of diagnosis (hospital vs ambulatory care) were evaluated [42] (Additional file 1: Table 5). Baseline haemoglobin ≥ 10 g/dL (HR 0.2, 95% CI 0.1–0.6) [43] and culture conversion in patients with XDR-TB, with final follow up sputum as conversion (HR 0.1, 95% CI 0.1–0.3) or reversion (HR 0.2, 95% CI 0.1–0.5) had a protective effect on mortality [17] (Additional file 1: Table 5).

#### Treatment-related factors

Drugs and regimens used for treating DR-TB were considered in five studies [17, 28, 29, 32, 35]. The use of ethambutol in XDR-TB treatment regimens among PLHIV was associated with an increased risk of mortality (HR 3.1, 95% CI 1.0–9.7) [17]. A significant protective effect was reported for increasing the number of drugs included in XDR-TB regimens (HR 0.6, 95% CI 0.5–0.8) [32] as well as the use of specific drugs (clofazimine and moxifloxacin) (Additional file 1: Table 6).

#### HIV-related factors

HIV status (independent of ART) was reported as a risk factor in six studies [17, 28–30, 33, 46]. The greatest risk of mortality was reported in miners living with HIV not on ART (aIRR 3.6, 95% CI 1.9–6.7 for death in the first month of TB treatment and aIRR 7.8, 95% CI 5.2 to 11.8 2–6 months after starting TB treatment) [41]. In addition, co-infected adults with XDR-TB (aOR 2.9, 95% CI 1.3–6.3 [29]) or MDR-TB (HR 1.9, 95%CI 1.0–3.6) had an increased risk of mortality. For children with TBM, HIV was associated with an increased risk of mortality, but this effect was not significant in the final model when adjusted for drug resistance (aOR 6.2, 95% CI 0.9–41.3) [46]. CD4 count was evaluated as a risk factor in eight studies [18, 30, 35, 38, 40, 42, 44, 49] and found to be associated with mortality in six [18, 30, 38, 40, 42, 44]. Three of these studies analysed CD4 count as a continuous variable and reported increased risk of death as CD4 counts fell. The other three studies considered CD4 count categorically and the greatest effect was reported in adults living with HIV with CD4 < 100 cells/mm^3^ compared to CD4 > 100 cells/mm^3^ (aOR 18.0, 95% CI 1.5–210.6) [38]. Two studies reported the effect of HIV viral load [42, 49] with viral suppression (< 5 log copies/ml) associated with decreased risk of mortality in children with HIV (HR 0.4, 95% CI 0.2–0.9) [49]. In adults with XDR-TB, initiating ART was associated with reduced risk of mortality in three studies (HR 0.1, 95% CI 0.0–0.5 [17], aHR 0.3, *p* value = 0.009 [18], and HR 0.4, 95% CI 0.2–0.8 [32]). Not being on ART was associated with increased risk of death compared to HIV-negative individuals (OR 2.5, 95% CI 1.0 to 6.3) [28]. Timing of ART was evaluated in patients with MDR-TB and initiating ART before initiating TB treatment was associated with increased risk of mortality (OR 1.7, CI 95% 1.0–2.7) [35] (Additional file 1: Table 7).

## Discussion

Despite TB being preventable, treatable, and curable, in this systematic review we found that one in four South African patients with a TB diagnosis died. This is higher than the 15% (or one in seven) reported by the WHO for 2018 (in their most recent estimates of TB burden, generated for the Global Tuberculosis Report), because WHO numbers reflect estimates for all incident TB whereas our review is restricted to those on TB treatment, and because the WHO estimate includes deaths prior to TB notification and reflects deaths over a one-year period whereas our review includes some studies with greater periods of follow up and specific populations with higher risk of mortality [2, 50].

In our review, the risk of mortality was highest among patients with DR-TB, where death was observed in more than a third of all patients. In contrast, in those diagnosed with DS-TB, the risk was lower, with death observed in one in eight patients. Risk of mortality was equal for PLHIV and HIV-negative TB patients, with death observed in one in five patients in each group; however, this is because the pooled CFR for the HIV-negative group is based primarily on studies of DR-TB patients.

Individual studies included in this review indicate that HIV remains a major risk factor for TB mortality. This is in line with findings from previous studies as well as a national study evaluating treatment outcomes of all TB patients started on treatment, reporting that patients with HIV on ART had a greater hazard of death compared to patients without HIV, and those not on ART had two-threefold increased risk of mortality compared to patients who were HIV-negative [23, 24, 51]. In our systematic review, the protective effect of ART on TB mortality relates to patients treated between 1995 and 2012, a period when ART was not available for all PLHIV, including TB patients. Earlier work has shown that unknown HIV status is associated with increased risk of mortality compared to a negative status [8, 51], possibly as PLHIV had an unknown status and were not accessing ART. Our study findings are aligned with a meta-analysis reporting that ART during TB treatment reduced mortality by 44–71% [52]. In addition to ART timing, our study emphasizes the important protective role of viral suppression on ART against mortality; the extremely high CFR reported among hospitalised HIV-positive individuals with low CD4 counts < 350 cells/mm^3^ from suspected bacterial sepsis and disseminated TB, underscores the impact of failure to timeously diagnose and initiate treatment in patients for both TB and HIV, and possibly delayed patient health seeking behavior. In South Africa, the response to treating HIV and TB has shifted considerably with all PLHIV eligible for ART as of 2016 [53]. This increased access to ART is likely to reduce mortality in TB patients, but studies in our review did not cover this period.

Previous TB treatment was reported as a risk factor in two studies we reviewed [28, 32]. Studies of routine data have also shown significant associations between previous treatment history and increased TB mortality [8, 23, 24, 51], suggesting this may relate to the proportion of undiagnosed DR-TB in patients with previous TB treatment [8], a higher likelihood of extensive lung damage, and previous TB episode-related co-morbidities [51]. Drug regimens administered to TB patients may impact the risk of mortality, but these effects are unclear. As all DR-TB treatment outcomes were reported for 2015 or earlier, our review did not include the use of bedaquiline, delamanid, or newer shorter regimens for DR-TB but indicated protective effects for moxifloxacin, clofazimine, and the number of drugs included in XDR-TB treatment regimens [32]. These results have been confirmed in patients with HIV and DR-TB, treated with at least one WHO DR-TB Group A drug (moxifloxacin, levofloxacin, bedaquiline, or linezolid), who were at a significantly lower risk of death compared to those not treated with a group A drug [54]. Ethambutol was reported to have a threefold increased risk of mortality in the small sample of XDR patients with HIV [17]; however, this is likely reflective of a period where patients had limited treatment options rather than the effect of the drug.

Multiple studies reported advancing age increasing the risk of mortality in adults [28, 33, 41, 44]; especially for those over 50 years. This is comparable to studies conducted on smaller segments of the South African population [8, 51] as well as the study of the National TB treatment register for DS-TB [24] and indicates that older patients may have differing treatment needs. This is also reflective of changing trends in the profile of people dying from TB in South Africa, with mortality decreasing in those under 50 years, but increasing in older patients [55]. Although mixed results for sex and mortality [8, 24, 51, 56] are reported, results from the analyses of the National TB treatment register and mortality registrations indicate that men are more likely to die from TB than women [24]. While this may reflect biological differences, it is in part attributed to women’s higher participation in HIV-related services [24, 55], better adherence to TB treatment and lower risk of LTFU [51].

A strength of this systematic review is that we included studies with moderate to large samples of TB patients that were reasonably representative of target study populations; that used medical records and other objective measures to establish exposure and outcomes; and sampled comparison groups from comparable sources. Multiple factors compromised the quality of the evidence, including the lack of prospective studies and RCTs, and the reliance on statistical procedures rather than sampling or design strategies to control for potential confounders. This increased the potential for unknown systematic bias. Inconsistency in risk factors and comparator variables made it difficult to assess the quality for each of the outcomes. Variances could not be pooled and estimated across studies, and we were unable to perform a meta-analysis of risk factors for TB mortality. A further limitation is that collectively the studies in this review do not accurately represent the population of people with TB in South Africa. In this review, the proportion of patients with DR-TB or living with HIV was much higher than estimated for South Africa and the proportion of children was under-represented. In our review 41% of TB patients included were miners in South Africa, but we had insufficient data to evaluate additional risk factors for mortality in this population such as silicosis. This review did not provide sufficient data to consider co-morbidities and final causes of death, nor was there sufficient data relating to sociostructural determinants of health such as those related to socioeconomic status, healthcare system access and quality and behavioural factors (e.g. cigarette smoking). Finally, this study evaluated studies published between 2010 and 2018 and included data on patients treated between 1995 and 2015. We acknowledge that this does not include earlier studies which may inform the results, nor does it include studies which address the impact of COVID-19 on TB mortality.

## Conclusions

Introducing standardised variables and minimum reporting requirements for TB cohorts would support future comparative work. We recommend at a minimum including sex; age with WHO age bands as a minimum; weight, including BMI; HIV status including HIV unknown, negative, and positive with an indication of ART regimen and timing; immune suppression with CD4 counts; diagnostic tests used with DST; and the place or level of care where the diagnosis was made. Published articles should include explicit statements about follow-up and the duration of follow-up and should include all WHO TB treatment outcome categories with descriptors of those who are LTFU. While sex was not identified as a definitive risk factor for TB mortality, specific interventions which target improvements in case finding and retention in care should focus on the differing needs of males and females. Based on existing evidence it is important to further examine the impact of factors such as pregnancy, diabetes mellitus, heart disease, chronic lung disease, and malignancy, which are associated with increased TB mortality [6, 57–60]. Further, we note the limited data on TB mortality and risk factors for mortality in children and adolescents indicating the importance of further studies of these groups. Additional studies on how sociostructural determinants of health impact TB mortality outcomes are also needed. Finally, given the likely impact of COVID-19 on TB and TB mortality, we recommend that an additional review be conducted to examine this.

## Supplementary Information

**Additional file 1:** Supplementary material. Description of data: Complete list of outcomes and variables for which data were sought; PRISMA 2020 checklist. **Supplementary Table 1.** Newcastle-Ottawa quality assessment scale for cohort studies, South Africa, 2010-2018 (*n*=21). **Supplementary Table 2.** Cochrane risk of bias tool for randomised trials, South Africa, 2010-2018 (*n*=2). **Supplementary Table 3.** Newcastle-Ottawa quality assessment scale for case control studies, South Africa, 2010-2018 (*n*=1). **Supplementary Table 4.** Demographic risk factors for TB mortality. **Supplementary Table 5.** Clinical risk factors for TB mortality, South Africa, 2010-2018. **Supplementary Table 6.** Tuberculosis treatment-related risk factors for TB mortality, South Africa, 2010-2018. **Supplementary Table 7.** HIV and antiretroviral therapy related risk factors for TB mortality, South Africa, 2010-2018.

## Data Availability

The data generated or analysed during this study are included in this published article and its supplementary information files.

## Abbreviations

ART: Antiretroviral therapy
CFR: Case fatality ratio
CI: Confidence interval
DR: Drug-resistant
DS: Drug-susceptible
DST: Drug-susceptible test
HR: Hazard ratio
IRR: Incident rate ratio
LTFU: loss to follow-up
MDR: Multidrug-resistant
NOS: Newcastle-Ottawa scale
OR: Odds ratio
PLHIV: People living with HIV
RCT: Randomised control trial
RR: Relative risk
TB: Tuberculosis
TBM: Tuberculosis meningitis
u-LAM: Urinary lipoarabinomannan
WHO: World Health Organization
XDR: Extensively drug-resistant
