# Supplementary material for: A systematic review of risk factors for mortality among tuberculosis patients in South Africa
Source: Syst Rev. 2023 Feb 23;12:23. doi: 10.1186/s13643-023-02175-8 (PMC9946877; doi:10.1186/s13643-023-02175-8)
Supplement: Supplementary file 1 — Additional file 1: Supplementary material. Description of data: Complete list of outcomes and variables for which data were sought; PRISMA 2020 checklist. Supplementary Table 1. Newcastle-Ottawa quality assessment scale for cohort studies, South Africa, 2010-2018 (n=21). Supplementary Table 2. Cochrane risk of bias tool for randomised trials, South Africa, 2010-2018 (n=2). Supplementary Table 3. Newcastle-Ottawa quality assessment scale for case control studies, South Africa, 2010-2018 (n=1). Supplementary Table 4. Demographic risk factors for TB mortality. Supplementary Table 5. Clinical risk factors for TB mortality, South Africa, 2010-2018. Supplementary Table 6. Tuberculosis treatment-related risk factors for TB mortality, South Africa, 2010-2018. Supplementary Table 7. HIV and antiretroviral therapy related risk factors for TB mortality, South Africa, 2010-2018. [file 13643_2023_2175_MOESM1_ESM.docx]

**Supplementary material**

**Title**

A systematic review of risk factors for mortality among tuberculosis patients in South Africa

**Authors**

Tamaryn J Nicholson (1), Graeme Hoddinott (1), James A Seddon (1,2), Mareli M Claassens (1,3), Marieke M van der Zalm (1), Elisa Lopez (1,4), Peter Bock (1), Judy Caldwell (5), Dawood Da Costa (6), Celeste de Vaal (7), Rory Dunbar (1), Karen Du Preez (1), Anneke C Hesseling (1), Kay Joseph (5), Ebrahim Kriel (8), Marian Loveday (9, 10), Florian M Marx (1, 11), Sue-Ann Meehan (1), Susan Purchase (1), Kogieleum Naidoo (10), Lenny Naidoo (5), Fadelah Solomon-Da Costa (5), Rosa Sloot (1), Muhammad Osman (1, 12)

**Affiliations**

1. Desmond Tutu TB Centre, Department of Paediatrics and Child Health, Faculty of Medicine and Health Sciences, Stellenbosch University, Cape Town, South Africa
2. Department of Infectious Diseases, Imperial College London, London, United Kingdom
3. Department of Human, Biological and Translational Medical Sciences, School of Medicine, University of Namibia, Windhoek, Namibia
4. IS Global, Barcelona Centre for International Health Research (CRESIB), Hospital Clínic - Universidad de Barcelona, Barcelona, Spain
5. Community Services and Health Directorate, City of Cape Town, South Africa
6. Division of Medical Microbiology, Department of Pathology, Faculty of Medicine and Health Sciences, Stellenbosch University and National Health Laboratory Service, Tygerberg Hospital, Cape Town, South Africa
7. Division of Forensic Medicine and Toxicology, Department of Pathology, Faculty Health Sciences, University of Cape Town, South Africa
8. Metro Health Services, Southern and Western Substructure, Western Cape Government: Health, Cape Town, South Africa
9. HIV and other Infectious Diseases Prevention Research Unit, South African Medical Research Council, Durban, KwaZulu-Natal, South Africa
10. Centre for the AIDS Programme of Research in South Africa, CAPRISA-SA-MRC HIV-TB Pathogenesis and Treatment Research Unit, Nelson R Mandela School of Medicine, University of KwaZulu-Natal, Durban, South Africa
11. DSI-NRF Centre of Excellence in Epidemiological Modelling and Analysis (SACEMA), Stellenbosch University, Stellenbosch, South Africa
12. School of Human Sciences, Faculty of Education, Health and Human Sciences, University of Greenwich, London, United Kingdom

**Complete list of outcomes and variables for which data were sought**

**Main outcomes:** the primary outcome for analysis will be risk factors for TB-related mortality as indicated by effect measures (e.g. odds or hazard ratios). The WHO defines TB death as "all-cause mortality before completing anti-TB treatment" as TB-associated death is hard to establish with confidence^[[1]](#footnote-2)^. This definition was used for this review.

**Additional outcomes:** secondary outcomes will include:1. Risk factors for mortality in drug-susceptible versus drug-resistant tuberculosis, 2. Risk factors for mortality in paediatric versus adult patients, 3. Risk factors for mortality in HIV infected versus HIV uninfected patients, 4. Descriptors of contextual risk factors including a) location, for example: home versus facility, and b) timing, for example: timing of death, documented by time to death from diagnosis and/or treatment initiation.

**Extracted data:**

| 1. Unique identifying number of the paper, 2. Identifying author 3. Date 4. Confirmation that the country in which the study was conducted is South Africa 5. Tuberculosis type (MDR or DS) 6. Population of interest 7. Sample size 8. Sampling strategy, 9. TB death or case-fatality rate definition used in the study 10. Case-fatality rate 11. Duration of follow-up post-treatment initiation 12. Treatment type (drugs named) 13. Length of treatment | 1. Identified risk factors for mortality and their effect estimates (e.g. odds or hazard ratios) and 95% confidence intervals or alternative statistical data 2. Place of death 3. Time between death and treatment initiation 4. Time between diagnosis and death 5. Year of TB treatment initiation 6. Diagnosis type (for example: clinical, x-ray, laboratory-confirmed, postmortem) 7. Limitations listed by authors 8. Geographical location within South Africa at the provincial, district and city level where appropriate 9. Any notes particular to the article – these may be qualitatively relevant. |
| --- | --- |

| **Section and Topic** | **Item #** | **Checklist item** | **Location where item is reported** |
| --- | --- | --- | --- |
| **TITLE** | | |  |
| Title | 1 | Identify the report as a systematic review. | Title |
| **ABSTRACT** | | |  |
| Abstract | 2 | See the PRISMA 2020 for Abstracts checklist. | Abstract, pages 3-4 |
| **INTRODUCTION** | | |  |
| Rationale | 3 | Describe the rationale for the review in the context of existing knowledge. | Pages 5-6 |
| Objectives | 4 | Provide an explicit statement of the objective(s) or question(s) the review addresses. | Page 6 |
| **METHODS** | | |  |
| Eligibility criteria | 5 | Specify the inclusion and exclusion criteria for the review and how studies were grouped for the syntheses. | Pages 6-7 |
| Information sources | 6 | Specify all databases, registers, websites, organisations, reference lists and other sources searched or consulted to identify studies. Specify the date when each source was last searched or consulted. | Page 6 |
| Search strategy | 7 | Present the full search strategies for all databases, registers and websites, including any filters and limits used. | Page 6 |
| Selection process | 8 | Specify the methods used to decide whether a study met the inclusion criteria of the review, including how many reviewers screened each record and each report retrieved, whether they worked independently, and if applicable, details of automation tools used in the process. | Page 6 |
| Data collection process | 9 | Specify the methods used to collect data from reports, including how many reviewers collected data from each report, whether they worked independently, any processes for obtaining or confirming data from study investigators, and if applicable, details of automation tools used in the process. | Page 7 |
| Data items | 10a | List and define all outcomes for which data were sought. Specify whether all results that were compatible with each outcome domain in each study were sought (e.g. for all measures, time points, analyses), and if not, the methods used to decide which results to collect. | Additional file 1: referenced page 7 |
|  | 10b | List and define all other variables for which data were sought (e.g. participant and intervention characteristics, funding sources). Describe any assumptions made about any missing or unclear information. | Additional file 1: referenced page 7 |
| Study risk of bias assessment | 11 | Specify the methods used to assess risk of bias in the included studies, including details of the tool(s) used, how many reviewers assessed each study and whether they worked independently, and if applicable, details of automation tools used in the process. | Page 7 |
| Effect measures | 12 | Specify for each outcome the effect measure(s) (e.g. risk ratio, mean difference) used in the synthesis or presentation of results. | Page 8. Table 1. & Additional file 1 |
| Synthesis methods | 13a | Describe the processes used to decide which studies were eligible for each synthesis (e.g. tabulating the study intervention characteristics and comparing against the planned groups for each synthesis (item #5)). | Page 7 |
|  | 13b | Describe any methods required to prepare the data for presentation or synthesis, such as handling of missing summary statistics, or data conversions. | Page 7 |
|  | 13c | Describe any methods used to tabulate or visually display results of individual studies and syntheses. | NA – results tabulated. |
|  | 13d | Describe any methods used to synthesize results and provide a rationale for the choice(s). If meta-analysis was performed, describe the model(s), method(s) to identify the presence and extent of statistical heterogeneity, and software package(s) used. | NA – the meta-analysis for our primary aim could not be completed due to heterogeneity in effect measures and risk factors evaluated. Noted: page 7. |
|  | 13e | Describe any methods used to explore possible causes of heterogeneity among study results (e.g. subgroup analysis, meta-regression). |  |
|  | 13f | Describe any sensitivity analyses conducted to assess robustness of the synthesized results. |  |
| Reporting bias assessment | 14 | Describe any methods used to assess risk of bias due to missing results in a synthesis (arising from reporting biases). |  |
| Certainty assessment | 15 | Describe any methods used to assess certainty (or confidence) in the body of evidence for an outcome. |  |

| **RESULTS** | | |  |
| --- | --- | --- | --- |
| Study selection | 16a | Describe the results of the search and selection process, from the number of records identified in the search to the number of studies included in the review, ideally using a flow diagram. | Page 8 |
|  | 16b | Cite studies that might appear to meet the inclusion criteria, but which were excluded, and explain why they were excluded. | NA |
| Study characteristics | 17 | Cite each included study and present its characteristics. | Table 1 |
| Risk of bias in studies | 18 | Present assessments of risk of bias for each included study. | Additional file 1 |
| Results of individual studies | 19 | For all outcomes, present, for each study: (a) summary statistics for each group (where appropriate) and (b) an effect estimate and its precision (e.g. confidence/credible interval), ideally using structured tables or plots. | Figure 2&3; Table 1, 2 and 3. |
| Results of syntheses | 20a | For each synthesis, briefly summarise the characteristics and risk of bias among contributing studies. | Pages 7-14 |
|  | 20b | Present results of all statistical syntheses conducted. If meta-analysis was done, present for each the summary estimate and its precision (e.g. confidence/credible interval) and measures of statistical heterogeneity. If comparing groups, describe the direction of the effect. | Pages 7-14  NA – the meta-analysis for our primary aim could not be completed due to heterogeneity in effect measures and risk factors evaluated. Noted: page 7. |
|  | 20c | Present results of all investigations of possible causes of heterogeneity among study results. |  |
|  | 20d | Present results of all sensitivity analyses conducted to assess the robustness of the synthesized results. |  |
| Reporting biases | 21 | Present assessments of risk of bias due to missing results (arising from reporting biases) for each synthesis assessed. |  |
| Certainty of evidence | 22 | Present assessments of certainty (or confidence) in the body of evidence for each outcome assessed. |  |
| **DISCUSSION** | | |  |
| Discussion | 23a | Provide a general interpretation of the results in the context of other evidence. | Pages 14-17 |
|  | 23b | Discuss any limitations of the evidence included in the review. | Page 17 |
|  | 23c | Discuss any limitations of the review processes used. | Page 17 |
|  | 23d | Discuss implications of the results for practice, policy, and future research. | Pages 17- 18 |
| **OTHER INFORMATION** | | |  |
| Registration and protocol | 24a | Provide registration information for the review, including register name and registration number, or state that the review was not registered. | Abstract |
|  | 24b | Indicate where the review protocol can be accessed, or state that a protocol was not prepared. | Abstract |
|  | 24c | Describe and explain any amendments to information provided at registration or in the protocol. | Abstract: methods. |
| Support | 25 | Describe sources of financial or non-financial support for the review, and the role of the funders or sponsors in the review. | Abstract and Funding (page 20) |
| Competing interests | 26 | Declare any competing interests of review authors. | Page 20 |
| Availability of data, code and other materials | 27 | Report which of the following are publicly available and where they can be found: template data collection forms; data extracted from included studies; data used for all analyses; analytic code; any other materials used in the review. | Declarations (page 19) |

*From:*  Page MJ, McKenzie JE, Bossuyt PM, Boutron I, Hoffmann TC, Mulrow CD, et al. The PRISMA 2020 statement: an updated guideline for reporting systematic reviews. BMJ 2021;372:n71. doi: 10.1136/bmj.n71

For more information, visit: <http://www.prisma-statement.org/>

Supplementary Table 1. Newcastle-Ottawa quality assessment scale for cohort studies, South Africa, 2010-2018 (n=21)

|  |  | **First author and year published** | | | | | | | | | | |
| --- | --- | --- | --- | --- | --- | --- | --- | --- | --- | --- | --- | --- |
| Quality assessment criteria | Acceptability | Lawn 2017 | Marais 2014 | Umanah 2015 | Field 2014 | Olaleye 2016 | Kerkhoff 2016 | Griesel 2018 | Marais 2011 | Janssen 2017 | Pietersen 2014 | Brust 2017 |
| **Selection ^a^** | | | | | | | | | | | | |
| Cohort representativeness | Cohort was truly or somewhat representative of people with DS-/MDR-/ XDR-TB in the community. | ***** | ***** | ***** | ***** | ***** | ***** | ***** | ***** | **-** | ***** | ***** |
| Selection of non-exposed cohort | Same community as the intervention cohort. | ***** | ***** | ***** | ***** | ***** | ***** | ***** | ***** | ***** | ***** | ***** |
| Ascertainment of intervention/ exposure | Secure record or structured interview. | ***** | ***** | ***** | ***** | ***** | ***** | ***** | ***** | ***** | ***** | ***** |
| **Comparability** | | | | | | | | | | | | |
| Study controls for main factors? | TB-type, HIV-status, age, sex. ^b^ | ***** | ***** | ***** | ***** | ***** | ***** | ***** | ***** | ***** | ***** | ***** |
| Study controls for additional factors? | Any two or more of the following: TB-type, HIV-status, age, sex, site of disease, TB treatment, prior TB treatment, weight, ART, ART timing, TB treatment timing, default, loss to follow up, CD4+, viral load, immunosuppression, resistance, diagnostics (smear/radiograph), drug regimens etc. | ***** | ***** | ***** | ***** | ***** | ***** | ***** | **-** | ***** | ***** | ***** |
| **Outcomes** | | | | | | | | | | | | |
| Assessment of outcome | Record linkage or independent blind assessment. | ***** | ***** | ***** | ***** | ***** | ***** | ***** | ***** | ***** | ***** | ***** |
| Follow-up long enough for outcome to occur | Median follow-up >= 3 months | ***** | **-** | ***** | ***** | ***** | ***** | **-** | ***** | ***** | ***** | ***** |
| Adequacy of  follow-up of  cohorts | All subjects accounted for, or subjects lost to follow-up unlikely to introduce bias (number lost <=10% or description suggests that those lost are not systematically different. | ***** | ***** | ***** | ***** | **-** | ***** | ***** | ***** | ***** | ***** | ***** |
| **Overall Quality Score (Maximum = 8)** | | 8 | 7 | 8 | 8 | 7 | 8 | 7 | 6 | 7 | 8 | 6 |

| Quality assessment criteria | Acceptability | Pietersen 2015 | Kvasnovsky 2011 | Dheda 2010 | Pepper 2011 | Loveday 2012 | Yotebieng 2010 | Brust 2010 | Seddon 2012 | Kendon 2012 | O'Donnell 2013 |  |
| --- | --- | --- | --- | --- | --- | --- | --- | --- | --- | --- | --- | --- |
| **Selection ^a^** | | | | | | | | | | | | |
| Cohort representativeness | Cohort was truly or somewhat representative of people (Adults or Children) with DS-/MDR-/ XDR-TB in the community. | ***** | ***** | ***** | **-** | ***** | ***** | ***** | ***** | ***** | ***** |  |
| Selection of non-exposed cohort | Same community as the intervention cohort. | ***** | ***** | ***** | ***** | ***** | ***** | ***** | ***** | ***** | ***** |  |
| Ascertainment of intervention/ exposure | Secure record or structured interview. | ***** | ***** | ***** | ***** | ***** | ***** | ***** | ***** | ***** | ***** |  |
| **Comparability** | | | | | | | | | | | | |
| Study controls for main factors? | TB-type OR HIV-status OR age OR sex. ^b^ | ***** | ***** | ***** | ***** | **-** | **-** | ***** | ***** | ***** | ***** |  |
| Study controls for additional factors? | Any two or more of the following: TB-type, HIV-status, age, sex, site of disease, TB treatment, prior TB treatment, weight, ART, ART timing, TB treatment timing, default, loss to follow up, CD4+, viral load, immunosuppression, resistance, diagnostics (smear/ radiograph), drug regimens etc. | ***** | ***** | ***** | ***** | **-** | ***** | ***** | ***** | ***** | ***** |  |
| **Outcomes** | | | | | | | | | | | | |
| Assessment of outcome | Record linkage or independent blind assessment. | ***** | ***** | ***** | ***** | ***** | ***** | ***** | ***** | ***** | ***** |  |
| Follow-up long enough for outcome to occur | Median follow-up >= 3 months | **-** | ***** | ***** | ***** | ***** | ***** | ***** | ***** | ***** | ***** |  |
| Adequacy of  follow-up of  cohorts | All subjects accounted for, or subjects lost to follow-up unlikely to introduce bias (number lost <=10% or description suggests that those lost are not systematically different. | **-** | ***** | **-** | **-** | **-** | ***** | ***** | **-** | **-** | ***** |  |
| **Overall Quality Score (Maximum = 8)** | | 6 | 8 | 7 | 6 | 5 | 7 | 8 | 7 | 7 | 8 |  |

ART: Antiretroviral therapy, CFR: Case fatality ratio, DR: Drug-resistant, MDR: Multidrug-resistant, NOS: Newcastle-Ottawa scale, TB: Tuberculosis, XDR: Extensively drug-resistant; +: positive; -: negative

1. The NOS for cohort and case control studies typically comprises four questions relating to selection, with a maximum total of nine. Due to the nature of the outcomes under investigation, this item was not appropriate for assessing these studies and was therefore removed.
2. Main factors are dependent on the target population for each study, e.g. a study sampling from XDR patients would not need to control for TB-type but would need to control for other factors like HIV and ART initiation/ adherence.

Supplementary Table 2. Cochrane risk of bias tool for randomised trials, South Africa, 2010-2018 (n=2)

|  | Churchyard 2010 | Abdool Karim 2010 |
| --- | --- | --- |
| Domain 1: Risk of bias arising from the randomization process | | |
| 1.1 Was the allocation sequence random? | Y | Y |
| 1.2 Was the allocation sequence concealed until participants were enrolled and assigned to interventions? | PY | PY |
| 1.3 Did baseline differences between intervention groups suggest a problem with the randomization process? | N | PN |
| *Risk-of-bias judgement* | Low risk | Low risk |
| Domain 2: Risk of bias due to deviations from the intended interventions (effect of assignment to intervention) | | |
| 2.1 Were participants aware of their assigned intervention during the trial? | PY | Y |
| 2.2 Were carers and people delivering the interventions aware of participants' assigned intervention during the trial? | PY | Y |
| 2.3 If Y/PY/NI to 2.1 or 2.2: Were there deviations from the intended intervention that arose because of the trial context? | N | N |
| 2.4 If Y/PY to 2.3: Were these deviations likely to have affected the outcome? | - | - |
| 2.5 If Y/PY/NI to 2.4: Were these deviations from intended intervention balanced between groups? | - | - |
| 2.6 Was an appropriate analysis used to estimate the effect of assignment to intervention? | PY | Y |
| 2.7 If N/PN/NI to 2.6: Was there potential for a substantial impact (on the result) of the failure to analyse participants in the group to which they were randomized? | - | - |
| *Risk-of-bias judgement* | Low risk | Low risk |
| Domain 2: Risk of bias due to deviations from the intended interventions (effect of adherence to intervention) |  |  |
| 2.1 Were participants aware of their assigned intervention during the trial? | PY | Y |
| 2.2 Were carers and people delivering the interventions aware of participants' assigned intervention during the trial? | PY | Y |
| 2.3 – 2.6 Items not relevant for these studies. | - | - |
| *Risk-of-bias judgement* | Low risk | Low risk |
| Domain 3: Risk of bias due to missing outcome data | | |
| 3.1 Were data for this outcome available for all, or nearly all, participants randomized? | N | Y |
| 3.2 If N/PN/NI to 3.1: Is there evidence that the result was not biased by missing outcome data? | PN | - |
| 3.3 If N/PN to 3.2: Could missingness in the outcome depend on its true value? | N | - |
| 3.4 If Y/PY/NI to 3.3: Is it likely that missingness in the outcome depended on its true value? | - | - |
| *Risk-of-bias judgement* | Low risk | Low risk |
| Domain 4: Risk of bias in measurement of the outcome |  |  |
| 4.1 Was the method of measuring the outcome inappropriate? | N | N |
| 4.2 Could measurement or ascertainment of the outcome have differed between intervention groups? | PN | N |
| 4.3 If N/PN/NI to 4.1 and 4.2: Were outcome assessors aware of the intervention received by study participants? | N | PY |
| 4.4 If Y/PY/NI to 4.3: Could assessment of the outcome have been influenced by knowledge of intervention received? | - | N |
| 4.5 If Y/PY/NI to 4.4: Is it likely that assessment of the outcome was influenced by knowledge of intervention received? | - | - |
| *Risk-of-bias judgement* | Low risk | Low risk |
| Domain 5: Risk of bias in selection of the reported result |  |  |
| 5.1 Were the data that produced this result analysed in accordance with a pre-specified analysis plan that was finalized before unblinded outcome data were available for analysis? | PY | PY |
| Is the numerical result being assessed likely to have been selected, on the basis of the results, from... | | |
| 5.2. ... multiple eligible outcome measurements (e.g. scales, definitions, time points) within the outcome domain? | N | N |
| 5.3 ... multiple eligible analyses of the data? | PN | PN |
| *Risk-of-bias judgement* | Low risk | Low risk |

Y: Yes, PY: Probably yes, N: No, PN: probably no, NI: No information.

Supplementary Table 3. Newcastle-Ottawa quality assessment scale for case control studies, South Africa, 2010-2018 (n=1)

| Quality assessment criteria | Acceptability | Gandhi 2012 |
| --- | --- | --- |
| **Selection ^a^** | | |
| Adequate case definition | Yes, with independent validation. | **-** |
| Representativeness of cases | Consecutive or obviously representative series of cases. | **-** |
| Selection of controls | Community controls. | ***** |
| **Comparability** | | |
| Study controls for main factors ^b^ | TB-type OR HIV-status OR age OR sex. | ***** |
| Study controls for additional factors | Any two or more of the following, if not the same as those above: TB-type, HIV-status, age, sex, site of disease, TB treatment, prior TB treatment, weight, ART, ART timing, TB treatment timing, default, loss to follow up, CD4+, viral load, immunosuppression, resistance, diagnostics (smear/ radiograph), drug regimens etc. | ***** |
| **Exposure** | | |
| Ascertainment of exposure | Secure record or structured interview | ***** |
| Same method for cases/ controls | Yes | ***** |
| Non-response rate | Same rate for both groups. | ***** |
| **Overall Quality Score (Maximum = 8)** | | 6 |

ART: Antiretroviral therapy, DR: Drug-resistant, NOS: Newcastle-Ottawa scale, TB: Tuberculosis, +: positive; -: negative

1. The NOS for cohort and case control studies typically comprises four questions relating to selection, with a maximum total of nine. Due to the nature of the outcomes under investigation, this item was not appropriate for assessing these studies and was therefore removed.
2. Main factors are dependent on the target population for each study, e.g., a study sampling from XDR patients would not need to control for TB-type but would need to control for other factors like HIV and ART initiation/ adherence.

Supplementary Table 4. Demographic risk factors for TB mortality

| **Category** | **Risk factor** | **Comparator** | **TB** | **Study** | **Measure** | **Effect size** | **LL 95%** | **UL 95%** |
| --- | --- | --- | --- | --- | --- | --- | --- | --- |
| Age  (in years) | 25-42 | <25 | XDR | Kvasnovsky 2011 [1] | aOR | 3.5 | 1.3 | 9.6 |
|  | 55+ | <35 predicting death in 2-6 months | NS | Field 2014 [2] | aIRR | 2.68 | 1.66 | 4.33 |
|  | 55+ | <35 predicting early death | NS | Field 2014 [2] | aIRR | 2.43 | 1 | 5.88 |
|  | 42+ | < 25 | XDR | Kvasnovsky 2011 [1] | aOR | 2.2 | 0.8 | 6.5 |
|  | 60+ | 15-60 | MDR | Olaleye 2016 [3] | aHR | 2.05 | 1.24 | 5.64 |
|  | 45-54 | <35 predicting early death | NS | Field 2014 [2] | aIRR | 1.92 | 0.91 | 4.05 |
|  | 45-54 | <35 predicting death in 2-6 months | NS | Field 2014 [2] | aIRR | 1.77 | 1.11 | 2.68 |
|  | 35-44 | <35 predicting early death | NS | Field 2014 [2] | aIRR | 1.57 | 0.73 | 3.37 |
|  | 35-44 | <35 predicting death in 2-6 months | NS | Field 2014 [2] | aIRR | 1.53 | 1.01 | 2.33 |
|  | 26-35 | >55 | MDR | Brust 2018 [4] | HR | 1.21 | 0.27 | 5.41 |
|  | Continuous variable | | MDR | Loveday 2012 [5] | HR | 1.04 | 1.02 | 1.06 |
|  | 46-55 | >55 | MDR | Brust 2018 [4] | HR | 0.99 | 0.16 | 5.9 |
|  | Continuous variable | | XDR | Pietersen 2014 [6] | aHR | 0.98 | 0.95 | 1 |
|  | 36-45 | >55 | MDR | Brust 2018 [4] | HR | 0.74 | 0.15 | 3.73 |
|  | 36+ | 16-35 | MDR | Marais 2014 [7] | aOR | 0.59 | 0.28 | 1.24 |
|  | 1.5 - 2.9 | <1.5 | DS | Yotebieng 2010 [8] | HR | 0.32 | 0.12 | 0.88 |
|  | 3 - 4.9 | <1.5 | DS | Yotebieng 2010 [8] | HR | 0.28 | 0.09 | 0.83 |
|  | 5 -15 | <1.5 | DS | Yotebieng 2010 [8] | HR | 0.22 | 0.09 | 0.51 |
|  | 18-25 | >55 | MDR | Brust 2018 [4] | HR | 0.19 | 0.02 | 2.07 |
|  | 0-15 | 16-35 | MDR | Marais 2014 [7] | aOR | 0.18 | 0.02 | 1.8 |
| Sex  (DR-TB) | Male | Female | MDR | Umanah 2015 [9] | OR | 1.58 | 0.94 | 2.66 |
|  | Male | Female | MDR | Olaleye 2016 [3] | HR | 1.31 | 0.83 | 2.06 |
|  | Female | Male | MDR | Gandhi 2012 ([10] | aHR | 1.15 ^a^ |  |  |
|  | Female | Male | XDR | Gandhi 2012 ([10] | aHR | 1.12 ^a^ |  |  |
|  | Male | Female | XDR | Pietersen 2014 [6] | HR | 0.86 | 0.54 | 1.37 |
|  | Male | Female | MDR | Brust 2018 [4] | aHR | 0.82 | 0.35 | 1.92 |
|  | Male | Female | MDR | Marais 2014 [7] | OR | 0.62 | 0.30 | 1.29 |
| Sex (HIV+) | Male | Female | NS | Lawn 2017 [11] | aHR | 1.8 | 0.73 | 4.48 |
|  | Male | Female | MDR | Umanah 2015 [9] | OR | 1.58 | 0.94 | 2.66 |
| Sex (Children) | Male | Female | All | Seddon 2012 [12] | aOR | 0.38 | 0.06 | 1.68 |

aHR: adjusted hazard ratio, aOR: adjusted odds ratio; ART: Antiretroviral therapy, CFR: Case fatality ratio, DR: Drug-resistant, HR: hazard ratio, INH: Isoniazid, IRR: Incident rate ratio, MDR: multi drug-resistant; NS: Non-specified, OR: odds ratio; RIF: Rifampicin mono-resistant, TB: Tuberculosis, XDR: Extensively drug-resistant

1. Confidence intervals not provided but p-values reported >0.05

Supplementary Table 5. Clinical risk factors for TB mortality, South Africa, 2010-2018

| **Category** | **Risk factor** | **Comparator** | **TB** | **Study** | **Measure** | **Effect size** | **LL 95%** | **UL 95%** |
| --- | --- | --- | --- | --- | --- | --- | --- | --- |
| Weight | Continuous variable - increase |  | XDR | Kvasnovsky 2011 [1] | aOR | 0.9 | 0.9 | 1 |
| Weight  (HIV+) | Severely underweight (<16kg/m^2^) | Normal BMI (≥18.5–24.99kg/m^2^) | MDR | Umanah 2015 [9] | aOR | 3.71 | 1.89 | 7.29 |
|  | Underweight (≥16-18.49kg/m^2^) | Normal BMI (≥18.5–24.99kg/m^2^) | MDR | Umanah 2015 [9] | aOR | 2.35 | 1.3 | 4.26 |
|  | Overweight/Obese (25-29.99kg/m^2^) | Normal BMI (≥18.5–24.99kg/m^2^) | MDR | Umanah 2015 [9] | aOR | 2.3 | 0.92 | 5.71 |
| Weight  (HIV+ Children) | Weight for age z-score: <-3SD | ≥2SD | DS | Yotebieng 2010 [8] | HR | 6.22 | 2.21 | 17.54 |
|  | Weight for age z-score: -2 to -3SD | ≥2SD | DS | Yotebieng 2010 [8] | HR | 2.5 | 0.74 | 8.36 |
| Previous TB  (DS) | ≥1 previous TB episodes (death in first month) | First episode of TB | NS | Field 2014 [2] | aIRR | 2.2 | 1.49 | 3.26 |
|  | ≥1 previous TB episodes (death in months 2-6) | First episode of TB | NS | Field 2014 [2] | aIRR | 1.46 | 1.17 | 1.82 |
|  | History of previous TB | First episode of TB | DS, MDR | Marais 2011 [13] | OR | 1.1 | 0.46 | 2.40 |
| Previous TB  (DR) | Previous MDR treatment | No previous MDR TB treatment | XDR | Dheda 2010 [14] | aHR | 5.21 | 1.93 | 14.1 |
|  | Previous MDR episode | No previous MDR TB episode | XDR | Kvasnovsky 2011 [1] | aOR | 1.3 | 0.4 | 4.3 |
|  | Per month of previous treatment (continuous variable) | | XDR | Kvasnovsky 2011 [1] | aOR | 1 | 1 | 1.1 |
| Diagnostics | Possible TB diagnosis ^a^ (death in first month) | Confirmed TB diagnosis ^a^ | NS | Field 2014 [2] | aIRR | 6.28 | 3.18 | 12.43 |
|  | uLAM positive | uLAM negative | NS | Lawn 2017 [11] | aHR | 4.2 | 1.5 | 11.75 |
|  | Probable TB diagnosis ^a^ (death in first month) | Confirmed TB diagnosis ^a^ | NS | Field 2014 [2] | aIRR | 3.38 | 1.65 | 6.93 |
|  | Smear positive | Smear negative | MDR | Olaleye 2016 [3] | aHR | 3.29 | 2.11 | 5,57 |
|  | Smear positive | Not smear positive | MDR | Gandhi 2012 ([10] | aHR | 2.1 ^b^ |  |  |
|  | Smear positive at treatment start | Not specified | XDR | Kvasnovsky 2011 [1] | aOR | 2 | 1 | 4,1 |
|  | Cavitary lesions on radiograph | No cavitary lesions | MDR | Umanah 2015 [9] | aOR | 1.76 | 1.08 | 2.85 |
|  | Possible TB diagnosis ^a^ (death in months 2-6) | Confirmed TB diagnosis ^a^ | NS | Field 2014 [2] | aIRR | 1.73 | 1.3 | 2.3 |
|  | Probable TB diagnosis ^a^ (death in months 2-6) | Confirmed TB diagnosis ^a^ | NS | Field 2014 [2] | aIRR | 1.17 | 0.87 | 1.58 |
|  | Bilateral cavitary disease | Not bilateral cavitary disease | XDR | Kvasnovsky 2011 [1] | aOR | 1.1 | 0.5 | 2.4 |
|  | Smear positive | Not smear positive | XDR | Gandhi 2012 ([10] | aHR | 2.1 ^b^ |  |  |
|  | Screening at 6 months ^c^ | Screening at 12 months ^c^ | DS | Churchyard 2011 [15] | HR | 0.73 | 0.5 | 1.08 |
|  | Chest radiograph bilateral disease | Disease not bilateral | MDR | Brust 2018 [4] | HR | 0.61 | 0.23 | 1.64 |
|  | Screening at 6 months ^c^ (death within first 2 months) | Screening at 12 months ^c^ | DS | Churchyard 2011 [15] | HR | 0.48 | 0.23 | 0.98 |
| Resistance | MDR | Drug-susceptible TB | All | Seddon 2012 [12] | aOR | 63.9 | 4.84 | 843.2 |
|  | Resistant to RIF, INH, Streptomycin | Resistant to RIF and INH | MDR | Gandhi 2012 [10] | aHR | 2.45 |  |  |
|  | Resistant to RIF, INH, Ethambutol, Streptomycin | Resistant to RIF and INH | MDR | Gandhi 2012 [10] | aHR | 1.55 |  |  |
|  | Capreomycin rrs resistance A1401G mutation | Capreomycin rrs wildtype | XDR | Pietersen 2015 [16] | aOR | 0.59 | 0.21 | 1.65 |
| Resistance (HIV+) | RIF-resistant TB | No resistance | NS | Lawn 2017 [11] | aHR | 8.41 | 1.74 | 40.67 |
| TB site  (DS) | EPTB (death in months 2 – 6) | PTB | NS | Field 2014 [2] | aIRR | 0.57 | 0.42 | 0.77 |
|  | EPTB (death in first month) | PTB | NS | Field 2014 [2] | aIRR | 0.54 | 0.33 | 0.91 |
| TB site  (HIV+) | EPTB | PTB | NS | Kendon 2012 [17] | HR | 0.3 | 0.1 | 0.7 |
|  | EPTB & PTB | PTB | NS | Kendon 2012 [17] | HR | 0.3 | 0.1 | 0.6 |
| Conversion | Net reversion ^d^ (last sputum culture event reversion) | Not reversion ^d^ | XDR | Pietersen 2014 [6] | aHR | 0.24 | 0.12 | 0.48 |
|  | Net conversion ^d^ (last sputum culture event conversion) | Not conversion ^d^ | XDR | Pietersen 2014 [6] | aHR | 0.14 | 0.06 | 0.34 |
| TB strain | H Strain | LAM strain | XDR | Marais 2014 [7] | aOR | 5.11 | 1.81 | 14.4 |
|  | T Strain | LAM strain | XDR | Marais 2014 [7] | aOR | 1.72 | 0.61 | 4.85 |
|  | Beijing Strain | LAM strain | XDR | Marais 2014 [7] | aOR | 1.31 | 0.45 | 3.76 |
|  | Other strain | LAM strain | XDR | Marais 2014 [7] | aOR | 0.98 | 0.34 | 2.81 |
| Other | Ambulatory outpatient | Hospitalized inpatient | NS | Kerkhoff 2016 [18] | aHR | 3.27 | 1.2 | 8.93 |
|  | Renal impairment, GFR <60ml/min | GFR ≥60ml/min | NS | Kendon 2012 [17] | HR | 2.6 | 1.3 | 4.9 |
|  | Other opportunistic infections ^e^ | No other opportunistic infections ^e^ | MDR | Umanah 2015 [9] | aOR | 1.8 | 1.1 | 2.94 |
|  | Hepcidin level (per 10 ng/mL increase) (continuous variable) | | NS | Kerkhoff 2016 [18] | aHR | 1.1 | 1.1 | 1.17 |
|  | Adverse events ^f^ | No adverse events | MDR | Umanah 2015 [9] | aOR | 1.09 | 0.68 | 1.77 |
|  | Haemoglobin level (per g/DL decrease) (continuous variable) | | MDR | Umanah 2015 [9] | aOR | 0.9 | 0.79 | 1.02 |
|  | Mycobacteremia (in HIV+ patients) | No mycobacteremia | DS | Janssen 2017 [19] | aHR | 0.8 | 0.3 | 2.2 |
|  | Baseline haemoglobin (≥ 10 g/dL) | <10 g/dL) | NS | Kendon 2012 [17] | HR | 0.2 | 0.1 | 0.6 |

a: adjusted; ART: Antiretroviral therapy; CFR: Case fatality ratio; DR: Drug-resistant, HR: hazard ratio; INH: Isoniazid; IRR: Incident rate ratio; LL: lower limit of 95% confidence interval; MDR: multi drug-resistant; NS: Non-specified; OR: odds ratio; R: resistance; RIF: Rifampicin; TB: Tuberculosis; UL: upper limit of 95% confidence interval; XDR: Extensively drug-resistant

1. Confirmed if at least one sputum culture was positive for *Mycobacterium tuberculosis*; Probable if at least one sputum smear was positive for acid-fast bacilli; and Possible if there was at least one negative sputum culture and/or sputum smear
2. Confidence intervals not provided but p-values reported >0.05
3. Screening: All miners were routinely screened for TB using a miniature screening chest radiograph (100 mm x 100 mm)
4. Conversion defined as two consecutive negative sputum cultures at least 30 days apart and Reversion defined as two consecutive positive sputum cultures at least 30 days apart after initial sputum culture conversion.
5. Candidiasis, Pneumocystis jirovecii Pneumonia, Infection with Herpes Simplex Virus, Herpes zoster, Cryptococcus, Cytomegalovirus, Cervical Dysplasia or cancer, Kaposi's sarcoma or HIV-associated nephropathy
6. Ototoxicity, nephrotoxicity, hepatotoxicity, joint pains, gastro-intestinal symptoms like nausea and vomiting requiring treatment, visual changes and conjunctivitis, psychosis, depression, peripheral neuropathy, hyperuricemia, hypothyroidism, and gynaecomastia

Supplementary Table 6. Tuberculosis treatment-related risk factors for TB mortality, South Africa, 2010-2018

| **Category** | **Risk factor** | | **Comparator** | **TB** | **Study** | **Measure** | **Effect size** | **LL 95%** | **UL 95%** |
| --- | --- | --- | --- | --- | --- | --- | --- | --- | --- |
| Treatment  regimen (DR) | Ethambutol (HIV+) | No ethambutol | | XDR | Pietersen 2014 [6] | aHR | 3.12 | 1.01 | 9.67 |
|  | Co-amoxicillin/clavulanic acid | No Co-amoxicillin/clavulanic acid | | XDR | Pietersen 2015 [16] | aOR | 3.1 | 1.4 | 6.6 |
|  | Ofloxacin and moxifloxacin (HIV+ patients) | No ofloxacin and moxifloxacin | | XDR | Pietersen 2014 [6] | aHR | 2.7 | 0.78 | 9.32 |
|  | Effective treatment ^a^ | Not on effective treatment ^a^ | | XDR | Kvasnovsky 2011 [1] | aOR | 1.8 | 0.4 | 7.7 |
|  | Dapsone | No dapsone | | XDR | Dheda 2010 [14] | aHR | 1.79 | 0.84 | 3.85 |
|  | Ethambutol | No ethambutol | | XDR | Dheda 2010 [14] | aHR | 1.49 | 0.69 | 3.23 |
|  | Clarithromycin | No clarithromycin | | XDR | Dheda 2010 [14] | aHR | 1.46 | 0.61 | 3.52 |
|  | Terizidone | No terizidone | | XDR | Dheda 2010 [14] | aHR | 1.35 | 0.71 | 2.55 |
|  | Cotrimoxazole prophylaxis (HIV+) | No cotrimoxazole prophylaxis | | MDR | Umanah 2015 [9] | aOR | 1.3 | 0.47 | 3.54 |
|  | Isoniazid | No isoniazid | | XDR | Dheda 2010 [14] | aHR | 0.76 | 0.24 | 2.51 |
|  | Para-amino salicylic acid (HIV+ patients) | No para- amino salicylic acid | | XDR | Pietersen 2014 [6] | aHR | 0.68 | 0.14 | 3.36 |
|  | Number of drugs (continuous variable) |  | | XDR | Dheda 2010 [14] | aHR | 0.59 | 0.45 | 0.78 |
|  | Clofazamine | No clofazamine | | XDR | Dheda 2010 [14] | aHR | 0.54 | 0.14 | 2.08 |
|  | Azithromycin (HIV+ patients) | No Azithromycin | | XDR | Pietersen 2014 [6] | aHR | 0.53 | 0.11 | 2.62 |
|  | Moxifloxacin | No moxifloxacin | | XDR | Pietersen 2015 [16] | aOR | 0.39 | 0.14 | 1.05 |
|  | Clofazamine | No clofazamine | | XDR | Pietersen 2014 [6] | aHR | 0.38 | 0.16 | 0.87 |
|  | Capreomycin | No capreomycin | | XDR | Pietersen 2015 [16] | aOR | 0.27 | 0.04 | 1.67 |
|  | Moxifloxacin | No moxifloxacin | | XDR | Dheda 2010 [14] | aHR | 0.11 | 0.01 | 0.82 |

a: adjusted; DR: drug-resistant; HR: hazard ratio; LL: lower limit of 95% confidence interval; OR: odds ratio; UL: upper limit of 95% confidence interval; XDR: Extensively drug-resistant; +: positive

1. Effective treatment if they received at least 4 drugs to which their TB could be considered susceptible

Supplementary Table 7. HIV and antiretroviral therapy related risk factors for TB mortality, South Africa, 2010-2018

| **Category** | **Risk factor** |  | **Comparator** | **TB** | **Study** | **Measure** | **Effect size** | **LL 95%** | **UL 95%** |
| --- | --- | --- | --- | --- | --- | --- | --- | --- | --- |
| HIV status | Positive without ART (death in months 2-6) | | Negative or unknown | NS | Field 2014 [2] | aIRR | 7.8 | 5.15 | 11.8 |
|  | Positive | | Negative | All | Seddon 2012 [12] | aOR | 6.17 | 0.92 | 41.30 |
|  | Positive | | Negative | DS, MDR | Marais 2011 [13] | OR | 4.1 | 0.48 | 34.38 |
|  | Positive without ART (death in first month) | | Negative or unknown | NS | Field 2014 [2] | aIRR | 3.59 | 1.94 | 6.66 |
|  | Positive | | Negative | XDR | Pietersen 2015 [16] | aOR | 2.90 | 1.34 | 6.3 |
|  | Positive not on ART ^a^ | | Negative | XDR | Kvasnovsky 2011 [1] | aOR | 2.50 | 1.0 | 6.30 |
|  | Unknown | | Negative | MDR | Olaleye 2016 [3] | HR | 1.93 | 1.04 | 3.60 |
|  | Positive | | Negative | MDR | Olaleye 2016 [3] | aOR | 1.89 | 1.02 | 3.52 |
|  | Positive | | Negative | XDR | Pietersen 2014 [6] | aHR | 1.51 | 0.87 | 2.63 |
| CD4+ | <100 cells/mm^3^ | | CD4 100-200 cells/mm^3^ | DS, MDR | Pepper 2011 [20] | aOR | 18 | 1.55 | 210.62 |
|  | Time-varying ≤100 cells/mm^3^ | | HIV-negative | MDR | Brust 2018 [4] | aHR | 16.92 | 4.75 | 60.3 |
|  | ≤100 cells/mm^3^ | | HIV-negative | MDR | Brust 2018 [4] | aHR | 6.28 | 1.74 | 22.7 |
|  | Time-varying 100 -200 cells/mm^3^ | | HIV-negative | MDR | Brust 2018 [4] | aHR | 4.8 | 0.96 | 24.08 |
|  | <50 cells/mm^3^ | | >200 cells/mm^3^ | MDR | Gandhi 2012 [10] | aHR | 4.64 ^b^ |  |  |
|  | <50 cells/mm^3^ | | >200 cells/mm^3^ | XDR | Gandhi 2012 [10] | aHR | 4.46 ^b^ |  |  |
|  | 51-200 cells/mm^3^ | | >200 cells/mm^3^ | MDR | Gandhi 2012 [10] | aHR | 4.17 ^b^ |  |  |
|  | Immunosuppression (Severe)d | | No severe immunosuppression ^d^ | DS | Yotebieng 2010 [8] | HR | 2.38 | 0.85 | 6.68 |
|  | 51-200 cells/mm^3^ | | >200 cells/mm^3^ | XDR | Gandhi 2012 [10] | aHR | 2.34 ^c^ |  |  |
|  | 100 – 200 cells/mm^3^ | | HIV-negative | MDR | Brust 2018 [4] | aHR | 2.09 | 0.47 | 9.35 |
|  | <150 cells/mm^3^ | | ≥350 cells/mm^3^ | MDR | Umanah 2015 | OR | 1.56 | 0.73 | 3.38 |
|  | Continuous decrease per 50 units in CD4 cells/mm^3^ | | | DS, MDR | Marais 2011 [13] | OR | 1.4 | 1.03 | 1.96 |
|  | >200 cells/mm^3^ | | HIV-negative | MDR | Brust 2018 [4] | aHR | 1.15 | 0.27 | 4.84 |
|  | Continuous decrease per 10 units in CD4 cells/mm^3^ | | | NS | Kerkhoff 2016 | aHR | 1.13 | 1.04 | 1.23 |
|  | Time-varying > 200 cells/mm^3^ | | HIV-negative | MDR | Brust 2018 [4] | aHR | 0.65 | 0.15 | 2.75 |
|  | 150 – 349 cells/mm^3^ | | ≥350 cells/mm^3^ | MDR | Umanah 2015 [9] | OR | 0.63 | 0.28 | 1.42 |
|  | Continuous increase per 100 cells/mm^3^ | |  | NS | Griesel 2018 [21] | aOR | 0.62 | 0.45 | 0.86 |
| Viral load | Continuous per unit increase copies/mL | | | NS | Kerkhoff 2016 [18] | aHR | 0.69 | 0.47 | 1.02 |
|  | 0-5 log copies/mL | | >5 log copies/ml | DS | Yotebieng 2010 [8] | HR | 0.42 | 0.19 | 0.93 |
| ART | ART (death in months 2-6) | | Negative or unknown | NS | Field 2014 [2] | aIRR | 6.02 | 3.75 | 9.67 |
|  | Initiated as an inpatient | | Initiated as an outpatient | NS | Kendon 2012 | HR | 3.7 | 1.6 | 8.2 |
|  | ART (death in first month) | | Negative or unknown | NS | Field 2014 [2] | aIRR | 3.17 | 1.56 | 6.44 |
|  | Started before the start of TB treatment | | Started after the start of TB treatment | MDR | Umanah 2015 [9] | OR | 1.65 | 1.02 | 2.73 |
|  | Delayed ART >60 days | | Started ART ≤60 days | DS | Yotebieng 2010 [8] | aHR | 1.32 | 0.55 | 3.16 |
|  | Started early ^e^ | | Started immediately ^e^ | NS | Kendon 2012 [17] | HR | 1.1 | 0.4 | 3.2 |
|  | Delayed ART >30 days | | Started ART ≤30 days | DS | Yotebieng 2010 [8] | HR | 0.86 | 0.46 | 1.6 |
|  | Delayed ART >15 days | | Started ART ≤15 days | DS | Yotebieng 2010 [8] | HR | 0.82 | 0.48 | 1.41 |
|  | Received ART (time dependent) | | No ART | MDR | Gandhi 2012 [10] | aHR | 0.67 ^c^ |  |  |
|  | Integrated ART ^f^ | | Sequential ART ^f^ | NS | Abdool Karim 2010 [22] | HR | 0.43 | 0.25 | 0.79 |
|  | On ART | | HIV-positive patients not on ART | XDR | Dheda 2010 [14] | HR | 0.38 | 0.18 | 0.8 |
|  | Received ART (time dependent) | | No ART | XDR | Gandhi 2012 [10] | aHR | 0.34 ^b^ |  |  |
|  | Delayed start of ART ^e^ | | Started immediately ^e^ | NS | Kendon 2012 [17] | HR | 0.25 | 0.03 | 2 |
|  | On ART | | HIV-positive patients not on ART | XDR | Pietersen 2014 [6] | HR | 0.13 | 0.03 | 0.5 |

a: adjusted; ART: Antiretroviral therapy; DR: Drug-resistant, HR: hazard ratio; IRR: Incident rate ratio; LL: lower limit of 95% confidence interval; MDR: multi drug-resistant; NS: Non-specified; OR: odds ratio; TB: Tuberculosis; UL: upper limit of 95% confidence interval; XDR: Extensively drug-resistant

1. Reduced model restricted to HIV-positive patients not on ARVs and known HIV-negative patients
2. Confidence intervals not provided but p-values reported <0.05
3. Confidence intervals not provided but p-values reported >0.05
4. CD4%<25% in <1 years, CD4%<20% in <1-2 years, CD4%<15% in <3-15 years
5. The timing of ART initiation defined relative to TB treatment as : Immediate: TB patients who started ART ≤ 28 days after starting TB treatment; Early: TB patients who started ART 29-56 days after starting TB treatment; Delayed: TB patients who started ART ≥57 days after starting TB treatment [17]
6. The timing of ART initiation defined relative to TB treatment as: Integrated: where ART started within 4 weeks of starting TB treatment, or within 4 weeks of completing the intensive phase of TB treatment; and Sequential: ART: where ART was started within 4 weeks of completion of TB treatment [22]

**References**

1. Kvasnovsky CL, Cegielski JP, Erasmus R, Siwisa NO, Thomas K, der Walt ML. Extensively drug-resistant TB in Eastern Cape, South Africa: high mortality in HIV-negative and HIV-positive patients. J Acquir Immune Defic Syndr **2011**; 57(2): 146-52.

2. Field N, Lim MS, Murray J, Dowdeswell RJ, Glynn JR, Sonnenberg P. Timing, rates, and causes of death in a large South African tuberculosis programme. BMC Infect Dis **2014**; 14: 3858.

3. Olaleye AO, Beke AK. Survival of smear-positive multidrug resistant tuberculosis patients in Witbank, South Africa: A retrospective cohort study. Infect Dis **2016**; 48(6): 422-7.

4. Brust JCM, Shah NS, Mlisana K, et al. Improved Survival and Cure Rates With Concurrent Treatment for Multidrug-Resistant Tuberculosis-Human Immunodeficiency Virus Coinfection in South Africa. Clin Infect Dis **2018**; 66(8): 1246-53.

5. Loveday M, Wallengren K, Voce A, et al. Comparing early treatment outcomes of MDR-TB in decentralised and centralised settings in KwaZulu-Natal, South Africa. Int J Tuberc Lung Dis **2012**; 16(2): 209-15.

6. Pietersen E, Ignatius E, Streicher EM, et al. Long-term outcomes of patients with extensively drug-resistant tuberculosis in South Africa: a cohort study. Lancet **2014**; 383(9924): 1230-9.

7. Marais E, Mlambo CK, Lewis JJ, et al. Treatment outcomes of multidrug-resistant tuberculosis patients in Gauteng, South Africa. Infection **2014**; 42(2): 405-13.

8. Yotebieng M, Van Rie A, Moultrie H, et al. Effect on mortality and virological response of delaying antiretroviral therapy initiation in children receiving tuberculosis treatment. AIDS **2010**; 24(9): 1341-9.

9. Umanah T, Ncayiyana J, Padanilam X, Nyasulu PS. Treatment outcomes in multidrug resistant tuberculosis-human immunodeficiency virus Co-infected patients on anti-retroviral therapy at Sizwe Tropical Disease Hospital Johannesburg, South Africa. BMC Infect Dis **2015**; 15: 478.

10. Gandhi NR, Andrews JR, Brust JC, et al. Risk factors for mortality among MDR- and XDR-TB patients in a high HIV prevalence setting. Int J Tuberc Lung Dis **2012**; 16(1): 90-7.

11. Lawn SD, Kerkhoff AD, Burton R, et al. Diagnostic accuracy, incremental yield and prognostic value of Determine TB-LAM for routine diagnostic testing for tuberculosis in HIV-infected patients requiring acute hospital admission in South Africa: a prospective cohort. BMC Med **2017**; 15(1): 67.

12. Seddon JA, Visser DH, Bartens M, et al. Impact of drug resistance on clinical outcome in children with tuberculous meningitis. Pediatr Infect Dis J **2012**; 31(7): 711-6.

13. Marais S, Pepper DJ, Schutz C, Wilkinson RJ, Meintjes G. Presentation and outcome of tuberculous meningitis in a high HIV prevalence setting. PLoS One **2011**; 6(5): e20077.

14. Dheda K, Shean K, Zumla A, et al. Early treatment outcomes and HIV status of patients with extensively drug-resistant tuberculosis in South Africa: a retrospective cohort study. Lancet **2010**; 375(9728): 1798-807.

15. Churchyard GJ, Fielding K, Roux S, et al. Twelve-monthly versus six-monthly radiological screening for active case-finding of tuberculosis: a randomised controlled trial. Thorax **2011**; 66(2): 134-9.

16. Pietersen E, Peter J, Streicher E, et al. High frequency of resistance, lack of clinical benefit, and poor outcomes in capreomycin treated South African patients with extensively drug-resistant tuberculosis. PLoS One **2015**; 10(4): e0123655.

17. Kendon MA, Knight S, Ross A, Giddy J. Timing of antiretroviral therapy initiation in adults with HIV-associated tuberculosis: outcomes of therapy in an urban hospital in KwaZulu-Natal. S Afr Med J **2012**; 102(12): 931-5.

18. Kerkhoff AD, Meintjes G, Burton R, Vogt M, Wood R, Lawn SD. Relationship Between Blood Concentrations of Hepcidin and Anemia Severity, Mycobacterial Burden, and Mortality Among Patients With HIV-Associated Tuberculosis. J Infect Dis **2016**; 213(1): 61-70.

19. Jenkins HE, Yuen CM, Rodriguez CA, et al. Mortality in children diagnosed with tuberculosis: a systematic review and meta-analysis. Lancet Infect Dis **2017**; 17(3): 285-95.

20. Pepper DJ, Marais S, Wilkinson RJ, Bhaijee F, De Azevedo V, Meintjes G. Barriers to initiation of antiretrovirals during antituberculosis therapy in Africa. PLoS One **2011**; 6(5): e19484.

21. Griesel R, Stewart A, van der Plas H, Sikhondze W, Mendelson M, Maartens G. Prognostic indicators in the World Health Organization's algorithm for seriously ill HIV-infected inpatients with suspected tuberculosis. AIDS Res Ther **2018**; 15(1): 5.

22. Abdool Karim SS, Naidoo K, Grobler A, et al. Timing of initiation of antiretroviral drugs during tuberculosis therapy. N Engl J Med **2010**; 362(8): 697-706.

1. World Health Organization. Definitions and reporting framework for tuberculosis – 2013 revision (Updated December 2014 and

   Janary 2020). Geneva, Switzerland: WHO Press, **2020**. Report No.: 978 92 4 150534 5. [↑](#footnote-ref-2)
